# GSK3-β Stimulates Claspin Degradation via β-TrCP Ubiquitin Ligase and Alters Cancer Cell Survival

**DOI:** 10.3390/cancers11081073

**Published:** 2019-07-29

**Authors:** Elisa Cabrera, Prahlad Raninga, Kum Kum Khanna, Raimundo Freire

**Affiliations:** 1 Unidad de Investigación, Hospital Universitario de Canarias, Ofra s/n, La Cuesta, 38320 San Cristóbal de La Laguna, Tenerife, Spain; 2 Instituto de Tecnologías Biomédicas, Universidad de La Laguna, 38200 San Cristóbal de La Laguna, Tenerife, Spain; 3 QIMR Berghofer Medical Research Institute, Herston, QLD 4006, Australia; 4 Universidad Fernando Pessoa Canarias, 35450 Las Palmas de Gran Canaria, Spain

**Keywords:** Claspin, GSK3-β, DNA damage response, triple negative breast cancer, cancer treatment

## Abstract

Claspin is essential for activating the DNA damage checkpoint effector kinase Chk1, a target in oncotherapy. Claspin functions are tightly correlated to Claspin protein stability, regulated by ubiquitin-dependent proteasomal degradation. Here we identify Glycogen Synthase Kinase 3-β (GSK3-β) as a new regulator of Claspin stability. Interestingly, as Chk1, GSK3-β is a therapeutic target in cancer. GSK3-β inhibition or knockdown stabilizes Claspin, whereas a GSK3-β constitutively active form reduces Claspin protein levels by ubiquitination and proteasome-mediated degradation. Our results also suggest that GSK3-β modulates the interaction of Claspin with β-TrCP, a critical E3 ubiquitin ligase that regulates Claspin stability. Importantly, GSK3-β knock down increases Chk1 activation in response to DNA damage in a Claspin-dependent manner. Therefore, Chk1 activation could be a pro-survival mechanism that becomes activated upon GSK3-β inhibition. Importantly, treating triple negative breast cancer cell lines with Chk1 or GSK3-β inhibitors alone or in combination, demonstrates that Chk1/GSK3-β double inhibition restrains cell growth and triggers more apoptosis compared to individual treatments, thereby revealing novel possibilities for a combination therapy for cancer.

## 1. Introduction 

A correct control of DNA replication and the DNA damage response (DDR) is a key cellular mechanism to maintain genome integrity and prevent oncogenic transformation. Central in the DDR is the kinase ATR, that becomes activated upon a variety of DNA lesions, including ultraviolet light-induced damage, replication stress and double-strand breaks [1]. ATR transfers this activation signal to the downstream kinase Chk1, by phosphorylating Chk1 on Serines (Ser) 317 and 345. Activated Chk1, in turn, phosphorylates protein substrates that prevent genomic instability. For example, Chk1 inhibits cell cycle progression by phosphorylating and thereby inhibiting the action of different isoforms of Cdc25, activator phosphatases of cyclin-dependent kinase complexes [2].

Claspin is a central player in the ATR-Chk1 axis, and critical for both Chk1 activation in response to DNA damage and Chk1 inactivation during checkpoint recovery [3,4,5,6]. Claspin is also required for DNA replication under unperturbed conditions [7,8]. To achieve these functions, Claspin is heavily regulated, including by modifications by phosphorylation and (poly-)ubiquitination. As a consequence, Claspin protein levels oscillate during a normal cell cycle, increasing during S and G2 phases and decreasing during G1 and mitosis [3]. This control is, in part, due to the proteasomal dependent degradation induced by the E3 ubiquitin ligase anaphase-promoting complex/cyclosome (APC/C) associated with Cdh1 in G1 phase [9] and by the E3 ligase SCF^β-TrCP^, formed by Skp1, Cullin 1 and the F box containing protein Beta-transducin repeat-containing protein (β-TrCP), during mitosis [4,5,6]. Claspin degradation also occurs during checkpoint recovery when cells are released from the G2 checkpoint arrest [4,5,6]. Ubiquitination of Claspin by SCF^β-TrCP^ requires the binding of β-TrCP to a phospho-degron in Claspin (DSGxxS, amino acids 29–34). In this degron, Ser30 and Ser34 are phosphorylated in a manner that is dependent on the mitotic kinase Polo-like kinase 1 (Plk1) [4,5,6]. Several ubiquitin hydrolases, including USP7, USP29, USP20 and USP9X were described to stabilize Claspin by counteracting the polyubiquitination and proteasomal degradation, demonstrating a high complexity in the regulation of Claspin levels [10,11,12,13,14]. 

Many studies have shown dysregulation of the components of the ATR-Claspin-Chk1 pathway in different cancers [2,8]. However, as the activation of Chk1 and ATR promotes cell cycle arrest and DNA repair [15], inhibition of these kinases by small compounds was conceived as an opportunity to selectively sensitize tumour cells that heavily rely on this pathway for cell survival [16,17]. Indeed, in recent years several Chk1 inhibitors were developed and some of them are being tested in clinical trials. For example, the compound LY2606368 is currently used in a phase II clinical trial in patients with ovarian cancer, triple negative breast cancer or prostate cancer. 

In this work we describe a link between the ATR-Claspin-Chk1 pathway and Glycogen Synthase Kinase 3-β (GSK3-β). GSK3-β is one of the two isoforms described for GSK3- and in addition to modulating glycogen synthesis, it regulates many cellular processes, such as protein synthesis, cell proliferation, cell differentiation, neuronal signalling, immune function and inflammation [18]. Mechanistically, GSK3-β prefers a primed phosphorylation on Ser or threonine (Thr) residues located four amino acids upstream of the target residue in its substrates. For example, one of the first reported substrates for GSK3 was β-catenin [19]. β-catenin is first phosphorylated by Casein Kinase 1 (CK1) and subsequently by GSK3-β. The latter phosphorylation event creates a phospho-degron that is recognized by β-TrCP, thereby triggering polyubiquitination and degradation by the proteasome. GSK3-β was shown to promote the degradation of several proteins involved in proliferation, such as β-catenin, c-Myc, or Cyclin D [19,20,21] and is therefore associated with anti-tumour suppression activity. GSK3-β was additionally demonstrated to induce cell proliferation and survival in cancer, a function that is related with its ability to regulate the transcriptional regulator NF-κB [22,23]. 

GSK3-β expression was shown increased in multiple cancers including breast cancer and it correlates with poor prognosis in breast cancer patients [22]. Besides, GSK3-β inhibitors 9-ING-41 and 9-ING-87, can suppress the growth of breast cancer cells in vitro and in vivo, and can also sensitize breast cancer patient-derived tumour xenografts to a chemotherapeutic agent irinotecan [24]. Interestingly, 9-ING-41 is currently being tested in phase I/II clinical trial for advanced cancers including breast cancer. Moreover, increased Chk1 activity has been shown to correlate with radio- and chemo-resistance in multiple cancers and inhibition of Chk1 sensitizes colon cancer cells to cisplatin [25] and pancreatic cancer cells to gemcitabine [26]. In addition, Chk1 activation resulted in resistance to chemotherapeutic agents in non-small cell lung cancer cells, and its inhibition using AZD7762 abrogated such chemoresistance [27], suggesting that Chk1 activation may serve as an underlying resistance mechanism to chemotherapies and multiple targeted therapies. 

Here, we describe a link in which GSK3-β controls the ATR-Claspin-Chk1 pathway by regulating β-TrCP-mediated Claspin protein stability. Our findings show that inhibition of GSK3-β leads to activation of Chk1, a kinase involved in survival mechanisms for the cell, and therefore protective for cell death. Interestingly, we found that combined enzymatic inhibition of GSK3-β and Chk1 synergizes and triggers apoptosis in triple-negative breast cancer (TNBC) cells, and therefore provides an opportunity to make more effective combination therapy with GSK3-β inhibitors in TNBC and other cancers types.

## 2. Results

### 2.1. GSK3-β Modulates Claspin Protein Stability

**Figure 1:**
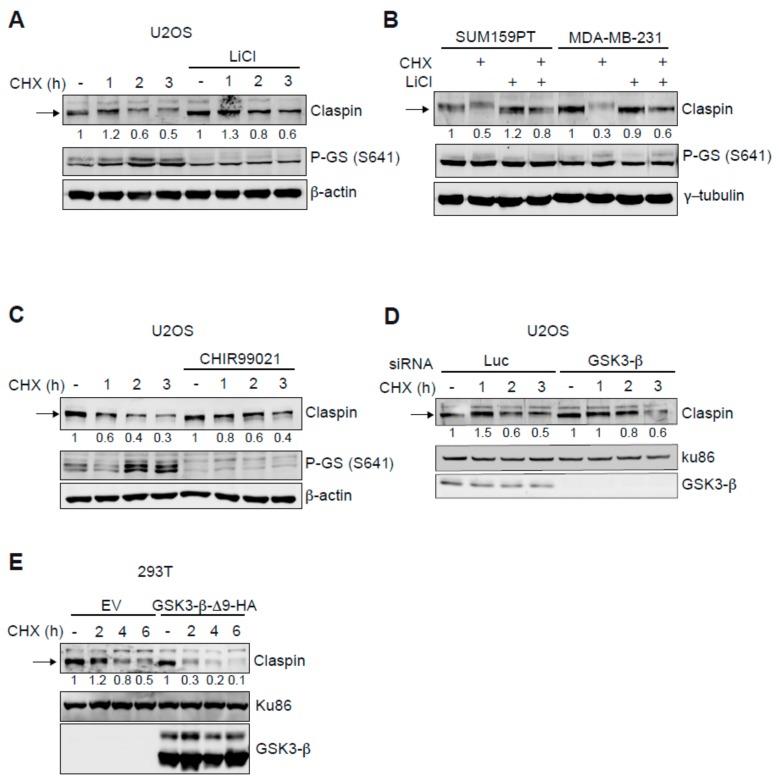
GSK3-β inhibition increases Claspin protein stability. U2OS (**A**) and TNBC cells SUM159PT and MDA-MB-231 (**B**) were incubated with lithium chloride (LiCl) or the GSK3-β inhibitor CHIR9901 (**C**) before cycloheximide (CHX, 35 µg/mL) addition for the indicated times before cell lysis. Analysis by immunoblot using the indicated proteins. (**D**) U2OS were downregulated for luciferase (Luc) or GSK3-β by siRNA for 48 h and then treated with cycloheximide (50 µg/mL) during the indicated times. Western blot analysis using the indicated antibodies. (**E**) 293T cells, transfected with empty vector (EV) or an expression plasmid for a constitutively active version of GSK3-β (∆9) were incubated with cycloheximide (50 μg/mL) for the indicated times before lysis. Shown is the western blot analysis for the indicated proteins. Ku86, γ-tubulin and β-actin and were used as loading controls. P-GS (S641) indicates the phosphorylated form of the Glycogen Synthase at Serine 641. Arrows indicate Claspin. Quantifications of Claspin levels are shown below each panel analysed and compared to loading control.

Claspin function in genome stability is modulated by its ubiquitin-dependent proteasomal degradation. Since ubiquitination can be regulated by phosphorylation, we searched for the existence of additional kinases that affect Claspin stability, by studying the effect of different kinase inhibitors available in our laboratory. Interestingly, although the incubation with some inhibitors reduced Claspin levels, lithium chloride treatment increased Claspin proteins levels in U2OS cells (Figure S1). To determine if these changes were due to protein stability, cells were incubated with the protein synthesis inhibitor cycloheximide for different times (Figure 1A). Indeed, treatment with lithium chloride leads to an increased Claspin stability. Lithium is a known inhibitor of the GSK3 family with a preference for the beta isoform (GSK3-β) [28,29]. As a positive control for inhibition GSK3-β by lithium, we checked for the phosphorylation of one of its substrates, Glycogen Synthase (GS) at Serine 641 (Figure 1A). We additionally examined the effect of lithium on Claspin in two TNBC cell lines, SUM159PT and MDA-MB-231. As shown in Figure 1B, addition of lithium increased Claspin protein stability in both cell lines. Although lithium chloride inhibits GSK3-β at high concentrations (millimolar range), it also inhibits other kinases. Therefore, to confirm that GSK3-β controls Claspin stability, CHIR99021, a potent and specific GSK3-β inhibitor [30], was used and also GSK3-β protein was downregulated by siRNA. Both treatment with CHIR99021 and siRNA-induced GSK3-β knock down resulted in Claspin stabilization (Figure 1C,D). As lack of GSK3-β activity is able to stabilize Claspin, increased GSK3-β activity should have the opposite response, and we therefore tested the effect of overexpression of a GSK3-β mutant lacking the first 9 amino acids (GSK3-β-∆9) and described to be constitutively active [31]. Indeed, expression of GSK3-β-∆9 reduced the half-life of Claspin (Figure 1E). Altogether these results indicate that GSK3-β is a new regulator of Claspin protein stability.

### 2.2. GSK3-β Interacts with Claspin and Regulates Claspin Ubiquitination

**Figure 2:**
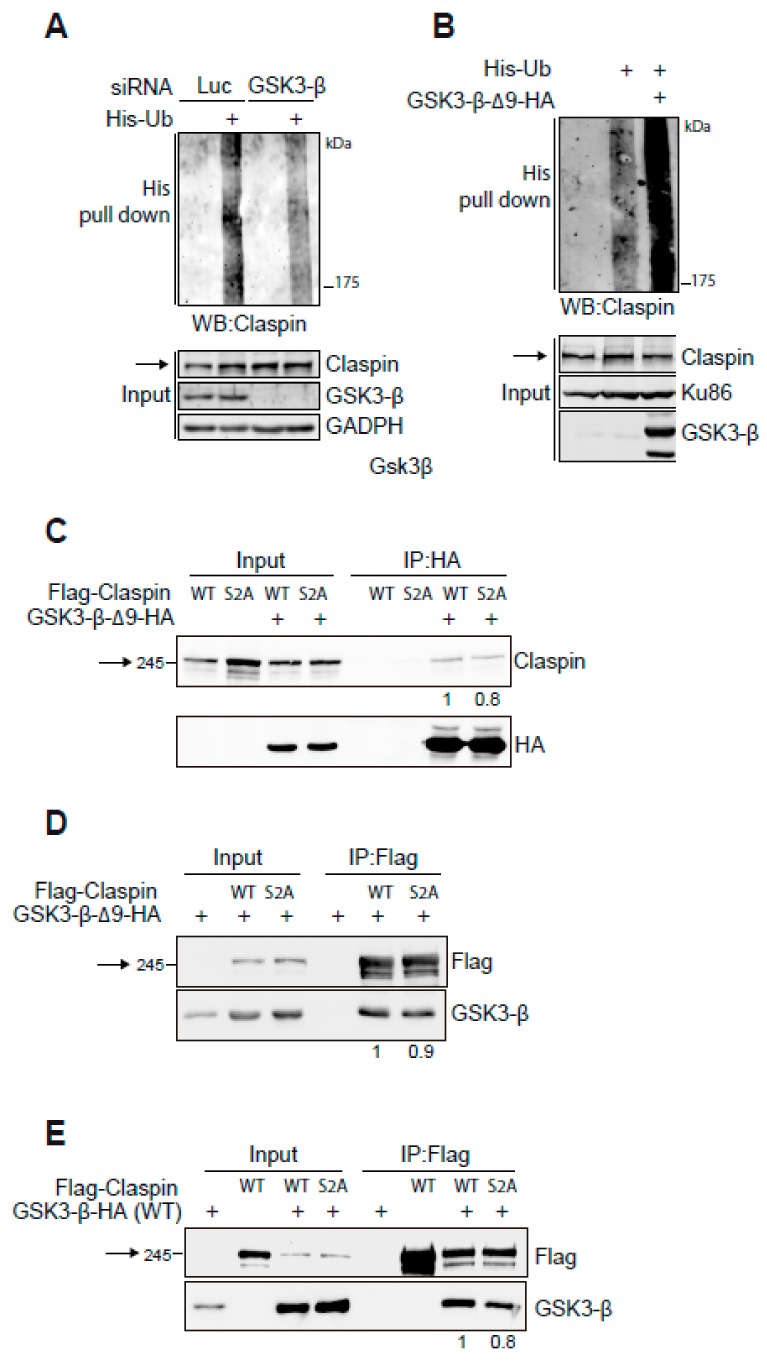
GSK3-β interacts with Claspin and promotes Claspin ubiquitination. (**A**) 293T, depleted for Luc or GSK3-β, were transfected with Flag-Claspin together with control or His-Ubiquitin plasmids. Cells were incubated with MG132 for 16 h before lysis under denaturing conditions. Western blot analysis of input and His-pulldowns were performed with the indicated antibodies. (**B**) Cells were transfected with Flag-Claspin, His-Ubiquitin and the GSK3-β-∆9 mutant and incubated with MG132 for 16 h. Then cells were lysed and analysed as in (**A**). (**C**,**D**) 293T cells, transfected with GSK3-β-Δ9-HA and the indicated versions of Flag-Claspin (WT; wild type, S2A: Ser30A/Ser34A mutant), were treated with MG132 for 16 h prior to lysis. Extracts were immunoprecipitated with anti-HA (**C**) or anti-Flag (**D**) beads and input and immunoprecipitates were analysed by Western blot with the indicated antibodies. (**E**) As (**D**) but using wild type GSK3-β-HA. Arrows indicate Claspin. Quantifications of the co-immunoprecipitated protein are shown below each panel analysed.

As Claspin levels are known to be controlled by ubiquitination and proteasomal degradation, we investigated if GSK3-β could promote Claspin ubiquitination. For this an in vivo ubiquitination assay was carried out in which Flag-Claspin and His-Ubiquitin were overexpressed, followed by a His pull-down assay and western blotting for Claspin. Upon GSK3-β depletion by siRNA, a decrease in Claspin polyubiquitination compared to cells transfected with control siRNA was observed (Figure 2A). In addition, overexpression of GSK3-β-∆9 strongly increased the polyubiquitination of Claspin (Figure 2B).

To test if the regulation of GSK3-β on Claspin was direct, interaction experiments by co-immunoprecipitation were performed. The GSK3-β consensus phosphorylation site is S/T-X-X-X-S^P^/T^P^ in which the last residue is prephosphorylated [32]. Since Claspin was described to contain a motif that is compatible with this consensus site and is related to its protein stability (amino acids 29-34, DSGQGS) [4,5,6,33], we performed the interaction experiment with both the wild type and Ser30Ala/Ser34Ala (S2A) mutant to study if this motif is involved in the interaction with GSK3-β. Although Claspin does not co-immunoprecipitate with the GSK3-β-∆9 mutant in normal conditions, upon stabilizing Claspin in the presence of MG132, an interaction between GSK3-β and Claspin could be detected in 293T, MDA-MB-231 and SUM159PT cells, suggesting a physical interaction between the two proteins (Figure S2A–C). Moreover, the interaction between the GSK3-β-∆9 mutant and both wild type and S2A versions of Claspin could be detected by performing immunoprecipitations of GSK3-β or Claspin (Figure 2C,D). Both versions of Claspin were shown to interact with GSK3-β wild type (Figure 2E). Although the interaction of GSK3-β with the Claspin S2A mutant was not abolished, it was slightly reduced, suggesting a putative mechanism of regulation of Claspin by GSK3-β.

### 2.3. Claspin-β-TrCP Interaction Is GSK3-β Dependent

**Figure 3:**
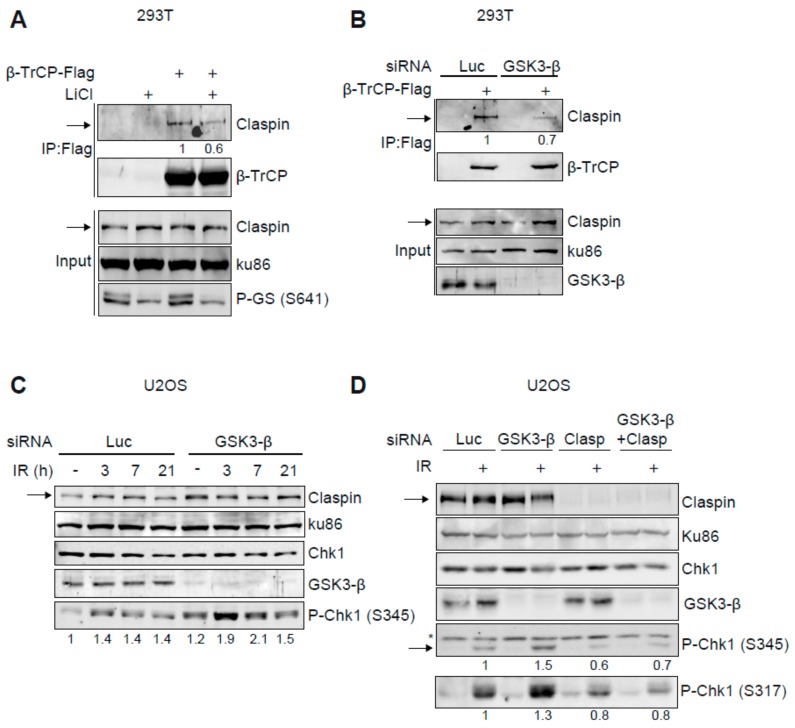
GSK3-β controls the Claspin-β-TrCP interaction and Chk1 activation. (**A**,**B**) 293T cells, transfected with HA-Claspin and β-TrCP-Flag were lysed, after which anti-Flag immunoprecipitations were analysed by Western blot with the indicated antibodies. In (**A**) cells were treated with 20 mM LiCl for 3 h before collection when indicated. In (**B**) 293T cells were depleted for Luc or GSK3-β by siRNA before plasmid transfection. (**C**) U2OS cells were downregulated for Luc or GSK3-β. 48 h post-transfection, cells were treated with IR (3Gy) and harvested at the indicated time points. Western blot analysis with the indicated antibodies. (**D**) U2OS cells were incubated with the indicated siRNAs for 48 h before irradiation (3Gy). 3 h after irradiation, extracts were made and analysed using the indicated antibodies. Ku86 was used as a loading control. P-Chk1 (Ser 317 or Ser345) stands for the phosphorylated forms of Chk1 on Serines 317 or 345, respectively. Arrows indicate Claspin, and in (**D**) also the specific band of Chk1 phosphorylated at Serine 345. Asterisk in (**D**) shows an aspecific band. Quantifications of phospo-Chk1 signal comparing to total Chk1 levels are shown below each panel analysed.

We next investigated how GSK3-β regulates Claspin stability. As SCF^β-TrCP^ is critical in controlling Claspin ubiquitination through the recognition of phosphorylated Claspin, we studied the impact of the kinase GSK3-β on the interaction between β-TrCP and Claspin. Claspin was readily detected in Flag-β-TrCP immunoprecipitates and treatment with lithium reduced the interaction of β-TrCP with Claspin (Figure 3A). Similarly, downregulation of GSK3-β by siRNA also reduced the co-immunoprecipitation of Claspin with β-TrCP (Figure 3B). Altogether, these data suggest that GSK3-β modulates the interaction of Claspin with β-TrCP and therefore Claspin polyubiquitination and subsequent degradation by the proteasome.

### 2.4. GSK3-β Regulates Chk1 Activation

Since Claspin is a crucial mediator protein in the activation of the DNA damage response effector kinase Chk1 and GSK3-β is able to modulate Claspin levels, we determined the impact of GSK3-β on activation of Chk1. In response to DNA damage, Chk1 activation is triggered by ATR-mediated phosphorylation of Ser345 (and Ser317). As shown in Figure 3C, depletion of GSK3-β by siRNA increased basal Chk1 phosphorylation in the absence of exogenous DNA damage (Figure 3C). In addition, after treating GSK3-β knock down cells with ionising radiation (IR), increased Chk1 phosphorylation was observed at early time points after IR and Chk1 phosphorylation remained high at later times as compared to control depleted cells (Figure 3C). To study if the increased activation of Chk1 occurring after GSK3-β inhibition was dependent on Claspin, both Claspin and GSK3-β were downregulated and Chk1 phosphorylation after DNA damage was studied. As shown in Figure 3D, the double knock down of Claspin and GSK3-β decreased Chk1 phosphorylation to levels similar to the ones observed with Claspin depletion only. Hence, our results suggest that GSK3-β inhibition increases Chk1 kinase activity in a Claspin-dependent fashion.

### 2.5. Combined Inhibition of GSK3-β and Chk1 Sensitizes Triple Negative Breast Cancer Cell Lines

**Figure 4:**
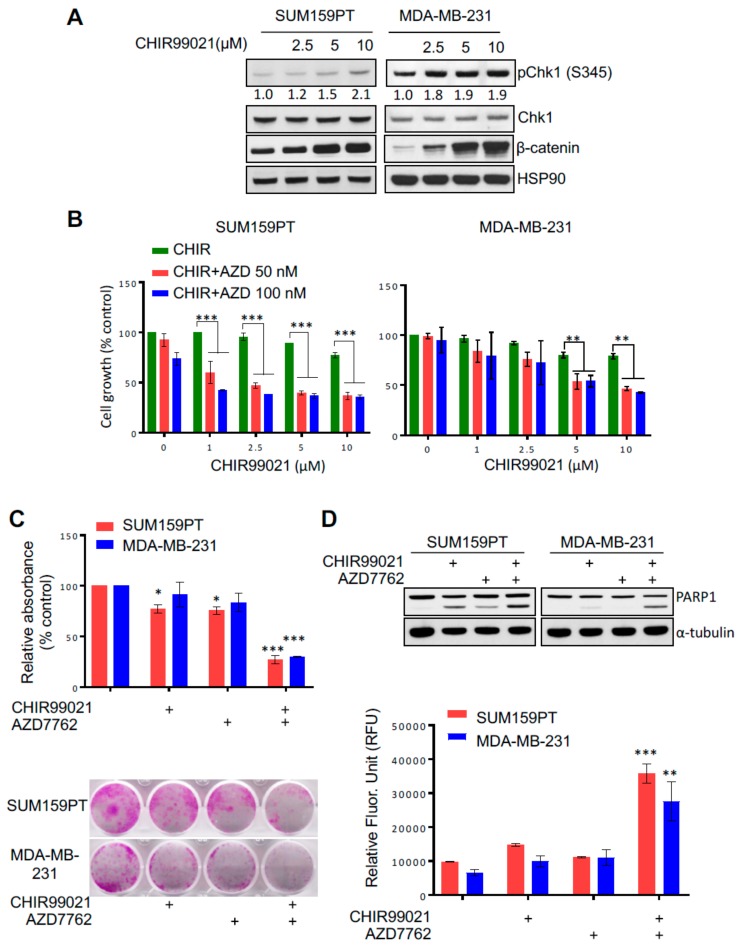
GSK3-β and Chk1 inhibition sensitizes triple negative cancer cell lines for apoptosis. (**A**) TNBC cells (SUM159PT and MDA-MB-231) were treated with the indicated concentrations of CHIR99021 for 24 h before collection and western blot analysis against the indicated antibodies. (**B**) When specified TNBC cells were treated with indicated concentrations of CHIR99021 and/or AZD7762 for 6 days. Cell proliferation was analysed by MTS assays. Two-way ANOVA followed by Tukey’s post-test were employed. (**C**,**D**) SUM159PT and MDA-MB-231 cells were treated with CHIR99021 (10 µM) and AZD7762 (100 nM) alone or in combination for 24 h. (**C**) Representative images of colony forming capacity (lower panel) of TNBC lines (SUM159PT and MDA-MB-231) following CHIR99021 and/or AZD7762 treatment at 14 days analysed using crystal violet staining. Quantification of the colonies formed in SUM159PT and MDA-MB-231 cells following indicated drug treatment measured by reading crystal violet absorbance (upper panel). One-way ANOVA followed by Bonferroni’s post-test were employed. Values indicate mean ± SD (*n* = 3). *, *p* < 0.05, ***, *p* < 0.001 (**D**) PARP1 cleavage (upper panel), and Caspase-3 activity (lower panel) in SUM159PT and MDA-MB-231 cells were measured following 24 h of drug treatment CHIR99021 and AZD7762 alone and in combination. Quantifications of phospo-Chk1 signal comparing to total Chk1 levels are shown below each panel analysed.

GSK3-β is a known oncotherapeutic target: inhibition GSK3-β with small compounds promotes cell death in different types of cells, including breast cancer [24,34,35]. Nevertheless, Claspin stabilization and Chk1 activation after GSK3-β inhibition might contribute to promoting survival, and thereby counteracting tumour removal. On the other hand, Chk1 kinase is also described to be a therapeutic target in cancer and increased Chk1 kinase activity has been shown to result in drug resistance [25,26,27]. Since GSK3-β knock down increased Chk1 phosphorylation and activation, we hypothesized that combined GSK3-β and Chk1 inhibition may exert synergistic anti-cancer activity. To investigate this hypothesis, two TNBC cell lines, SUM159PT and MDA-MB-231, particularly resistant to treatments, were chosen to study the effects of individual versus combined treatment with Chk1 (AZD7762) and GSK3-β inhibitors. As shown in Figure 4A, Chk1 phosphorylation was increased in both cell lines after treatment with the GSK3-β inhibitor. We also analysed β-catenin protein levels following CHIR99021 treatment in both TNBC lines as a surrogate marker for GSK3-β inhibition. Our data showed a marked increase in β-catenin protein levels, suggesting that CHIR99021 inhibited GSK3-β activity [19]. We next examined if Chk1 inhibition can sensitize TNBC cells to undergo cell death following GSK3-β inhibition. Indeed, co-treatment of SUM159PT and MDA-MB-231 cells with 50 nM and 100 nM Chk1 inhibitor AZD7762 sensitized both cells lines to the GSK3-β inhibitor CHIR99021 (Figure 4B). The MDA-MB-231 cell line is wild type for BRCA1 and PIK3CA, two genes often mutated in breast cancer, whereas the SUM159PT cell line contains wild type BRCA1 but also an activating PIK3CA mutation. To assess the influence of BRCA1 status on the proliferation after combined inhibition of Chk1 and GSK3-β, two extra cell lines were added to this study: MDA-MB-436 (BRCA1 mutant) and MDA-MB-468 (BRCA1 wild type). Figure S3 shows that the double inhibition of these two kinases inhibited the proliferation of both cell lines.

A decreased cell proliferation upon combined treatment was further confirmed in clonogenic survival assays, which demonstrated a reduction in colony formation after combined inhibition of Chk1 and GSK3-β (Figure 4C). Finally, to study if the growth defect was due to the induction of apoptosis, we examined PARP1 cleavage and caspase-3 activation, the classical markers of apoptosis, in SUM159PT and MDA-MB-231 cells following CHIR99021 and AZD7762 co-treatment. The combined treatment with both Chk1 and GSK3-β inhibitors dramatically increased the PARP1 cleavage in both TNBC lines (Figure 4D, top panel). In addition, co-treatment with CHIR99021 and AZD7762 significantly increased caspase-3 activity in both SUM159PT and MDA-MB-231 cells compared to a single agent treatment (Figure 4D, bottom panel). Altogether, these data show that Chk1 and GSK3-β inhibition cooperate to induce cell death in TNBCs and suggests a potential combination therapy for TNBCs.

### 2.6. Combination Treatment with GSK3-β and Chk1 Sensitizes Breast Cancer Cell Lines in 3D Cultures

**Figure 5:**
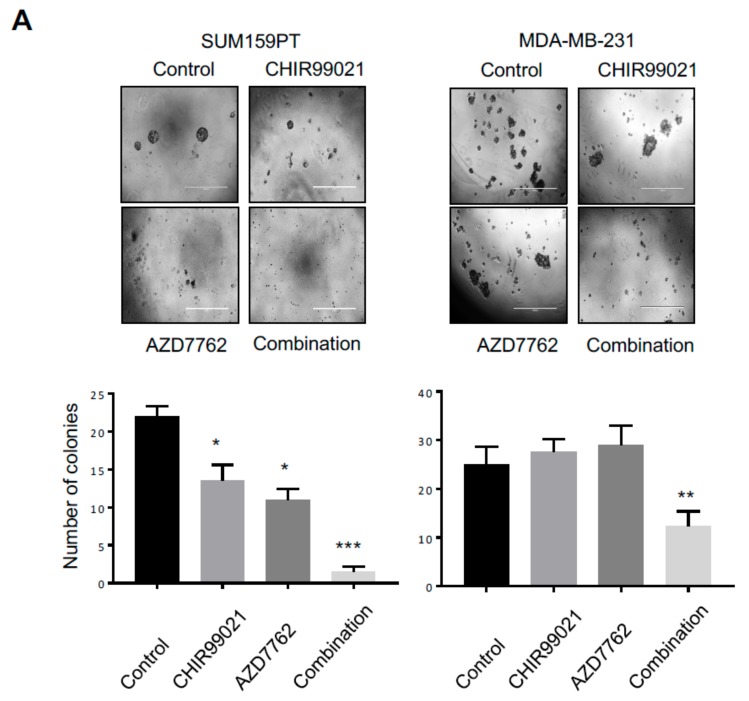

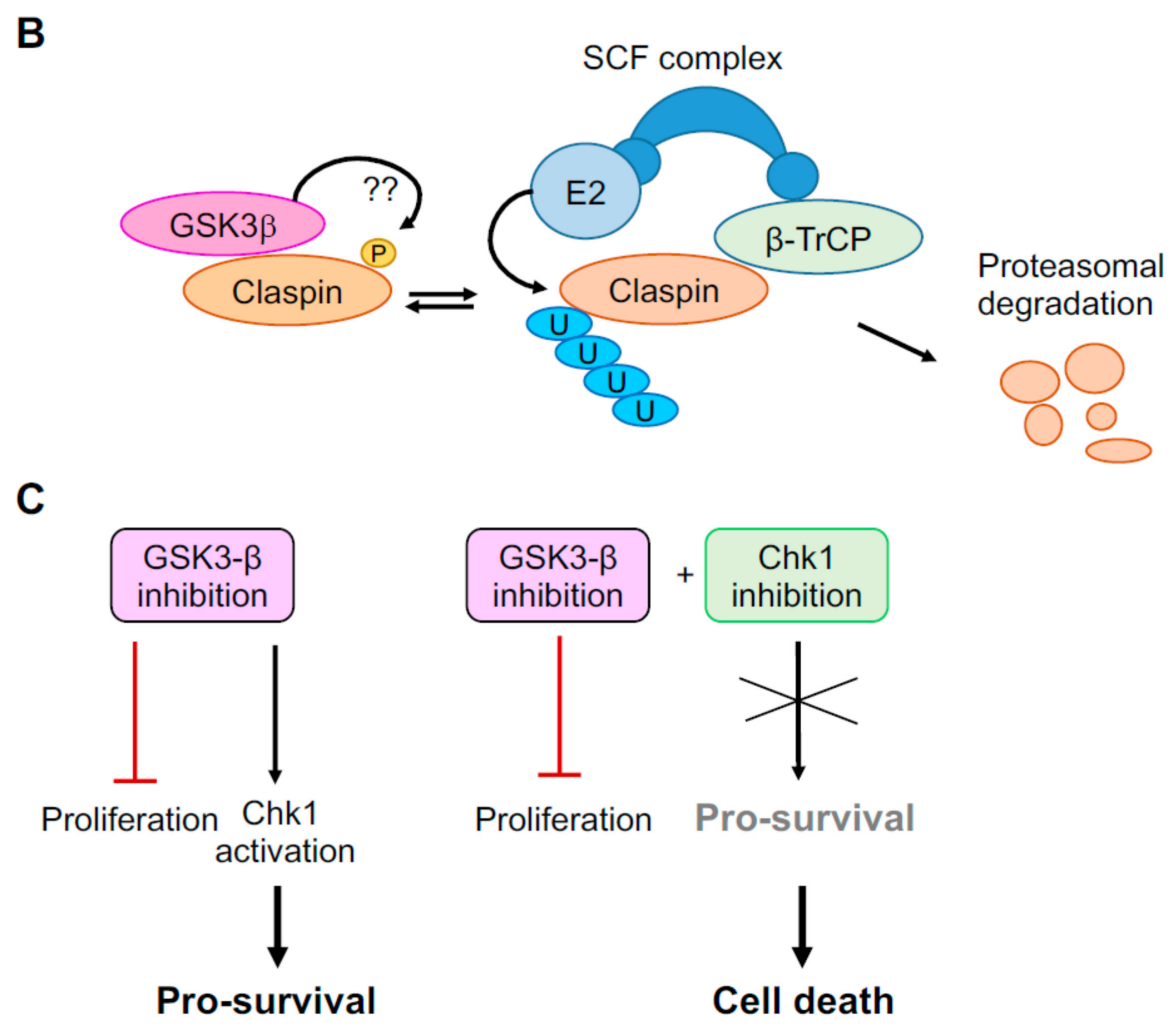
GSK3-β/Chk1 double inhibition reduces proliferation in three dimensional cultures. (**A**) Top panels, representative images of SUM159PT and MDA-MB-231 spheroids grown on Matrigel for 14 days treated with or without CHIR99021 (10 µM) and AZD7762 (100 nM) both alone and in combination. Bottom panels, quantification of a number of tumour spheroids treated with or without each treatment. One-way ANOVA followed by Bonferroni post-tests were employed. Values indicate mean ± SD (*n* = 3). *, *p* < 0.05, ** *p* < 0.01, ***, *p* < 0.001. (**B**) Schematic model on how GSK3-β can modulate Claspin stability. “U” represents ubiquitin and “P” represents phosphorylation. (**C**) Model on the GSK3-β and Chk1 inhibition on inhibiting proliferation and promoting cell death.

Next, the growth inhibitory effect of CHIR99021 and AZD7762 treatment both alone and in combination was evaluated on breast cancer lines, grown as spheroids in three dimensional (3D) cultures. Different to two dimensional (2D) cultures, spheroids mimic some aspects of solid tumours and are therefore a more valuable model for to evaluate the clinical relevance of a drug treatment. We observed that CHIR99021 and AZD7762 monotherapy reduced the number of SUM159PT, but not MDA-MB-231 spheroids. Notably, CHIR99021 and AZD7762 co-treatment significantly reduced the number of both SUM159PT and MDA-MB-231 spheroids (Figure 5A). Altogether, our data indicate that co-blockade of GSK3β and Chk1 significantly inhibits TNBC growth in vitro in both 2D and 3D cultures and suggest that in some tumours the double therapy could be effective.

## 3. Discussion

Here we identified the kinase GSK3-β as a new regulator of Claspin. Our data suggests that GSK3-β controls the ATR-Chk1 pathway in a negative way. The tight control of Claspin protein levels by ubiquitination and proteasome-dependent degradation, is critical for its cellular functions. Part of this control occurs at the mitotic onset in an unperturbed cell cycle but also when cells need to restart mitosis after the recovery from the DNA damage checkpoint when the DNA damage is repaired. In both these situations, Claspin is degraded in a Plk1- and SCF^β-TrCP^-dependent manner [4,5,6]. During this process, Claspin is phosphorylated at Ser 30 and 34, which creates a β-TrCP binding site leading subsequently to Claspin polyubiquitination and degradation.

Here we demonstrate that GSK3-β destabilizes Claspin protein and downregulation or enzymatic inhibition of GSK3-β increases Claspin half-life, by modulating Claspin polyubiquitination (Figure 1 and Figure 2A,B). Previously, GSK3-β was shown to regulate cellular processes by direct interaction with and phosphorylation of substrates but also by forming part of scaffold protein complexes that help GSK3-β accessing their substrates [36]. We demonstrate that Claspin interacts with GSK3-β in vivo (Figure 2C–E). The fact that the interaction is mildly reduced in the S2A mutant suggests that the interaction might be direct and that it is possible that phosphorylation of Ser34 might be involved. Moreover, GSK3-β affects the interaction of Claspin with β-TrCP, suggesting that GSK3-β creates of a phospho-degron in Claspin that is recognized by β-TrCP (Figure 3A,B and Figure 5B for a model). Unfortunately, we did not manage to determine if GSK3-β directly phosphorylates Ser30, so we cannot draw strict conclusions about the mechanism on how GSK3-β regulates Claspin stability. It is still possible that GSK3-β could destabilize Claspin using other phospho-degron sites and, accordingly, we noticed that Claspin might contain two potential phospho-degron sites. Indeed, amino acids 255-260 (ESGVHS) and 825-830 (SSGKLS) contain potential phospho-degron and phosphorylation site for GSK3-β. More experiments are needed to reveal the exact details of the molecular mechanism of regulation of Claspin stability by GSK3-β.

Our data show that inhibition of GSK3-β induces a stronger and more prolonged phosphorylation of checkpoint kinase Chk1 (Figure 3C). Interestingly, this activation is dependent on Claspin, as Claspin depletion abolishes this sustained Chk1 phosphorylation (Figure 3D). Chk1 activation promotes the G2/M cell cycle arrest and repair of the DNA damage [2]. This is a particularly important process in cancer cells that already are compromised for other DNA damage checkpoints, such as the G1/S checkpoint, by the mutation of proteins like p53. Consistent with this, Chk1 inhibition has been demonstrated to affect cell growth/proliferation of cancer cells [17]. Similarly, inhibition of GSK3-β represses cell viability in models of glioblastoma, leukaemia, ovarian, breast or prostate cancers [22]. However, if inhibition of GSK3-β activates Chk1 (Figure 3C), a GSK3-β inhibitor might be inhibiting cancer growth at the same time as promoting the pro-survival pathway ATR-Chk1. Therefore, we hypothesize that effectivity of GSK3-β inhibitors could be enhanced by concomitantly inhibiting Chk1 and such a combined inhibition of Chk1 and GSK3-β might be a more effective treatment against cancer cells (Figure 5C for a model). Interestingly, p53 pathway is mutated in >90% of triple negative breast cancers and both Chk1 and GSK3-β inhibitors have been shown to affect cell growth of cell lines derived from such tumours [24,34,35,37,38]. Importantly, we show that co-treatment with both Chk1 and GSK3-β inhibitors significantly reduces cell proliferation in four triple negative breast cancer cell lines with different mutations, whereas single treatments had only a minor effect. AZD7762 is a widely used and potent Chk1 inhibitor, however, because it also has some activity towards Chk2 we cannot formally rule out the possibility that inhibition of Chk2 might contribute to the synergistic cell killing observed. We consider this unlikely, however, since unlike Chk1, Chk2 is dispensable for cell proliferation and survival [39]. Indeed, the combination therapy reduced the proliferation regardless the BRCA1 and PIK3CA status (Figure 4B,C and Figure S3). Along with to the loss of cell proliferation we observed an increase in apoptosis (Figure 4D). Most significantly, the combination therapy was effective in 3D spheroid cultures, a closer model to solid tumours than 2D cultures. Taken together, our data suggests that the combined treatment with both Chk1 and GSK3-β inhibitors could be a novel effective therapy in triple negative cancers, but also possibly in other cancers in which inhibition of GSK3-β enhances Chk1 activation.

## 4. Materials and Methods

### 4.1. Cell Lines, Antibodies, Reagents and Plasmids

U2OS and 293T cells were grown using standard procedures. SUM159PT, MDA-MB-231, MDA-MB-468 and MDA-MB-436 cells were purchased from the American Type Culture Collection (ATCC, Baltimore, MD, USA) and maintained in DMEM media supplemented with 10% fetal bovine serum. Matrigel was purchased to Corning (Corning, Madrid, Spain). Commercial antibodies were as follows: Ku86 (C-20), Chk1 (G-4), GSK3-β (H-76), HSP90 (4F10), GAPDH (FL-335) from Santa Cruz Biotechnology (Santa Cruz Biotechnology, Heidelberg, Germany); pSer641-Glycogen Synthase (PA5-17702) from Thermo Fisher (Thermo Fisher Scientific, Madrid, Spain), β-actin (mouse monoclonal) from Genescript (Genescript, Piscataway, NJ, USA); pSer345-Chk1 (2341), PARP1 (9542S), β-catenin (D10A8) from Cell Signalling Technology (Danvers, MA, USA); and α-tubulin (T9026) and γ-tubulin (T6557) from Sigma-Aldrich (Madrid, Spain). Antiserum against Claspin and β-TrCP were previously described [4,40]. In Figure 1, CHIR99021 was used at 2 µM for 16 h and was purchased from Cayman Chemical Company (Tallinn, Estonia). MG132 was used at 5 µM for 16 h and was acquired from Calbiochem (San Diego, CA, USA). Lithium chloride (used at 20 mM), BIRB 796, UCN-01 and cycloheximide were purchased from Sigma-Aldrich and AZD7762 and most of the compounds used in the kinase screen (Figure S1) from Selleck Chemicals (Houston, TX, USA). HA-GSK-3β-∆9 (constitutively active) and HA-GSK-3β-WT expressing plasmids were obtained from A. Kikuchi (Hiroshima University, Hiroshima, Japan) and A. Cuadrado (Instituto de Investigaciones Biomédicas “Alberto Sols” UAM-CSIC, Madrid, Spain). An expression plasmid for His-Ubiquitin was a gift from D. Bohmann (Rochester, New York, NY, USA). HA-Claspin and Flag-β-TrCP expressing plasmids were described before [4].

### 4.2. Transfections

Plasmid transfections were performed using standard calcium phosphate method as described [41]. For downregulations, the following siRNA oligonucleotides purchased to Microsynth (Balgach, Switzerland) were used:

Luciferase5′-UCGAAGUAUUCCGCGUACGdTdT-3′GSK3-β5′-GCUAGAUCACUGUAACAUAdTdT-3′

Luciferase is not present in human cells and therefore can be used as a control for many experiments.

Cells were transfected with siRNAs using lipofectamine RNAiMax (Thermo Fisher) according to the manufacturer’s instructions.

### 4.3. Immunoprecipitations and In Vivo Ubiquitin Assays

Cells were lysed in a buffer containing 50 mM Tris-HCl, pH 8, 150 mM NaCl, 5 mM EDTA (pH 8), 0.5% NP-40, 5 mM NaF, 1 mM Na_3_VO_4_, 10 mM glycerolate and protease inhibitors (protease inhibitor cocktail set III, Calbiochem, San Diego, CA, USA). Immunoprecipitations were carried out with anti-Flag M2-agarose (Sigma-Aldrich) or with anti-HA antibody together with protein A-Sepharose CL-4B beads (GE Healthcare, Madrid, Spain) as previously described [41]. His-Ubiquitin pull downs were performed out using Nickel-NTA agarose (Qiagen, Hilden, Germany) as described before [4].

### 4.4. Irradiation

Cells were irradiated using a CellRad X-ray generator (Faxitron, Tucson, AZ, USA).

### 4.5. Cell Proliferation Assays

Cells were treated with CHIR99021 and AZD7762, both alone and in combination, for 6 days in 48-wells plate and cell viability was analysed by CellTiter 96^®^ AQueous One Solution Cell Proliferation Assay (MTS) (Promega, Madison, WI, USA) following the manufacturer’s guidelines.

### 4.6. Colony Formation Assays

Cells were treated with CHIR99021 and AZD7762 either alone or in combination for 24 h and effect of each treatment on long-term colony formation capacity were analysed as described previously [42].

### 4.7. Apoptotic Assays

Cells were treated with Chk1 and/or GSK3-β inhibitors for 24 h and apoptosis was analysed by measuring caspase-3 activity using a caspase-3 specific substrate Ac-DEVD-AMC as described previously [43].

### 4.8. Tumour Spheroid Assays

The three-dimensional tumour spheroid assays were performed using well established techniques as described previously [44]. Cells were stained with crystal violet and colonies were counted.

### 4.9. Western Blot Quantifications

Images were quantified using the ImageQuant TL software (GE Healthcare). 

## 5. Conclusions

We identified the kinase GSK3-β as a new regulator of the stability of Claspin, a DNA Damage Response mediator protein. GSK3-β promotes Claspin degradation via the proteasome and when GSK3-β is inhibited, Claspin is stabilized. GSK3-β modulates the interaction between Claspin and the F box protein β-TrCP, a critical component of the E3 ubiquitin ligase SCF^β-TrCP^. As a consequence of GSK3-β inhibition and Claspin stabilization, Chk1 is more active after DNA damage, thereby promoting survival of the cells. Therefore, GSK3-β inhibition as a therapy in cancer cells could be promoting undesired survival via Claspin stabilization and Chk1 activation. Here we show that inhibition of both GSK3-β and Chk1 are collaborating in promoting apoptosis in breast triple negative cancer cells, thereby opening new opportunities for cancer therapy.

## Supplementary Materials

The following are available online at https://www.mdpi.com/2072-6694/11/8/1073/s1, Figure S1: Effect of a panel of kinase inhibitors on Claspin protein levels, Figure S2: GSK3-β-Δ9 interaction with Claspin is detected after MG132 addition, Figure S3: GSK3-β and Chk1 double inhibition reduces the proliferation breast cancer cell regardless BRCA1 status.
