# Supplementary material for: GSK3-β Stimulates Claspin Degradation via β-TrCP Ubiquitin Ligase and Alters Cancer Cell Survival
Source: Cancers (Basel). 2019 Jul 29;11(8):1073. doi: 10.3390/cancers11081073 (PMC6721324; doi:10.3390/cancers11081073)
Supplement: Supplementary file 1 [file cancers-11-01073-s001.zip › cancers-565203-suppl-layout.docx]

Supplementary Materials

GSK3-β stimulates Claspin degradation via β-TrCP ubiquitin ligase and alters cancer cell survival

**Elisa Cabrera, Prahlad Raninga, Kum Kum Khanna and Raimundo Freire**


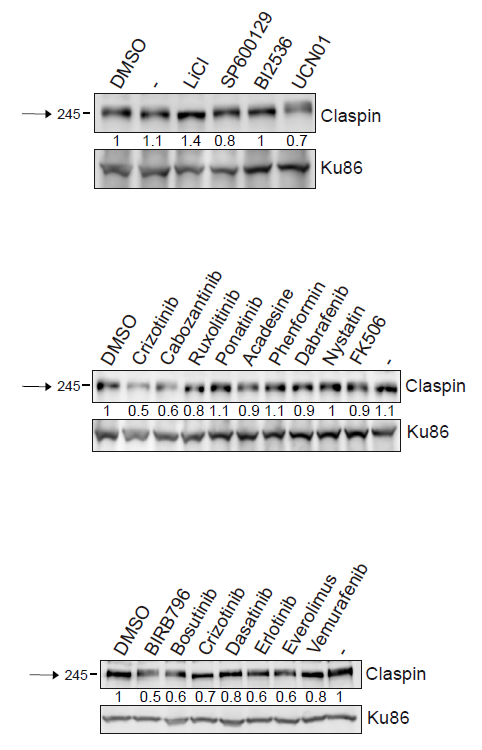


**Figure S1.** Effect of a panel of kinase inhibitors on Claspin protein levels. U2OS cells were treated for 2 h with the following compounds: LiCl (GSK3-β inhibitor), SP600129 (JNK inhibitor), BI2536 (PLK1 inhibitor) UCN01 (CHK1 inhibitor) or for 16 h with: Crizotinib, Cabozantinib (c-Met inhibitors), Ruxolitinib (JAK inhibitor), Ponatinib (Abl inhibitor), Acadesine, Phenformin (AMPK activators), Dabrafenib (Raf inhibitor), Nystatin (PI3K, Akt, and mTOR inhibitor), FK-506 (mTOR inhibitor), BIRB796 (p38 inhibitor), Bosutinib (Src and Abl inhibitor), Desatinib (Src, Bcr-Abl and c-Kit inhibitor), Erlotinib (EGFR inhibitor), Everolimus (mTOR inhibitor) and Vemurafenib (B-Raf inhibitor). All compounds were used at 10 µM except LiCl, used at 20 mM. After the incubation with the compounds, cell extracts were made and analysed using the indicated antibodies by immunoblot. Arrows indicate the Claspin band. Claspin levels quantifications are shown below each panel and compared to loading control (Ku86).


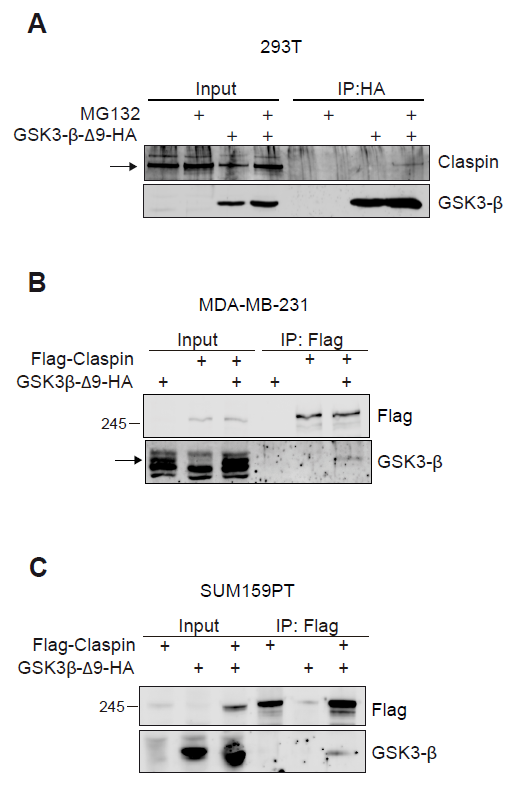


**Figure S2.** GSK3-β-Δ9 interaction with Claspin is detected after MG132 addition. 293T cells (**A**), MDA-MB-231 cells (**B**) or SUM159PT (**C**) were transfected with GSK3-β-Δ9-HA and Flag-Claspin. Prior to lysis, cells in (**A**) were treated, when indicated, with 5 µM MG132 for 16 h and in (**B**,**C**) all conditions were treated with 20 µM MG132 for 4 h. Anti-HA (**A**) or anti-Flag (**B**) immunoprecipitations were analysed by Western blot with anti-Claspin, anti-Flag and anti-GSK3-β antibodies. Arrows indicate the Claspin band, and in (**B**) also indicates the GSK3-β-Δ9 band.


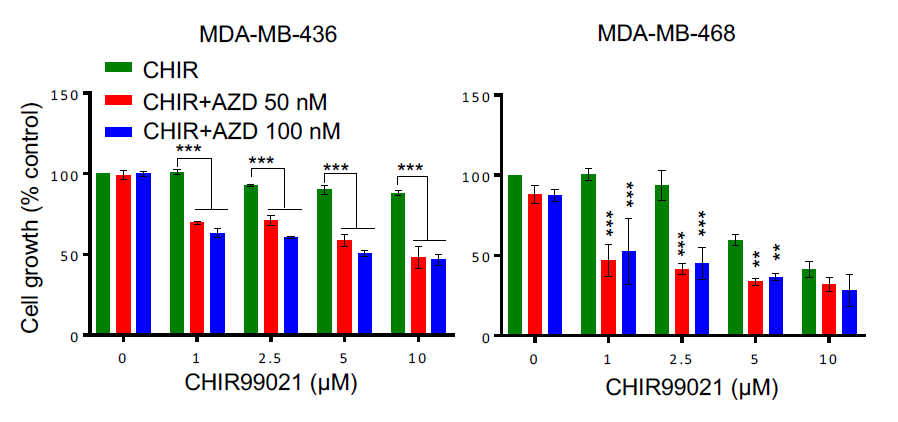


**Figure S3.** GSK3-β and Chk1 double inhibition reduces the proliferation breast cancer cell regardless BRCA1 status. When indicated, the MDA-MB-436 (BRCA1 mutant) and MDA-MB-468 (wild-type BRCA1) cell lines were treated with specified concentrations of CHIR99021 and/or AZD7762 for 6 days. Proliferation was analysed by MTS assays. Two-way ANOVA followed by Tukey’s post-test were employed. Values indicate mean ± SD (n = 3). **, *p* < 0.01, ***, *p* < 0.001.
