# Supplementary material for: GSK3-β Stimulates Claspin Degradation via β-TrCP Ubiquitin Ligase and Alters Cancer Cell Survival
Source: Cancers (Basel). 2019 Jul 29;11(8):1073. doi: 10.3390/cancers11081073 (PMC6721324; doi:10.3390/cancers11081073)

Figure 1

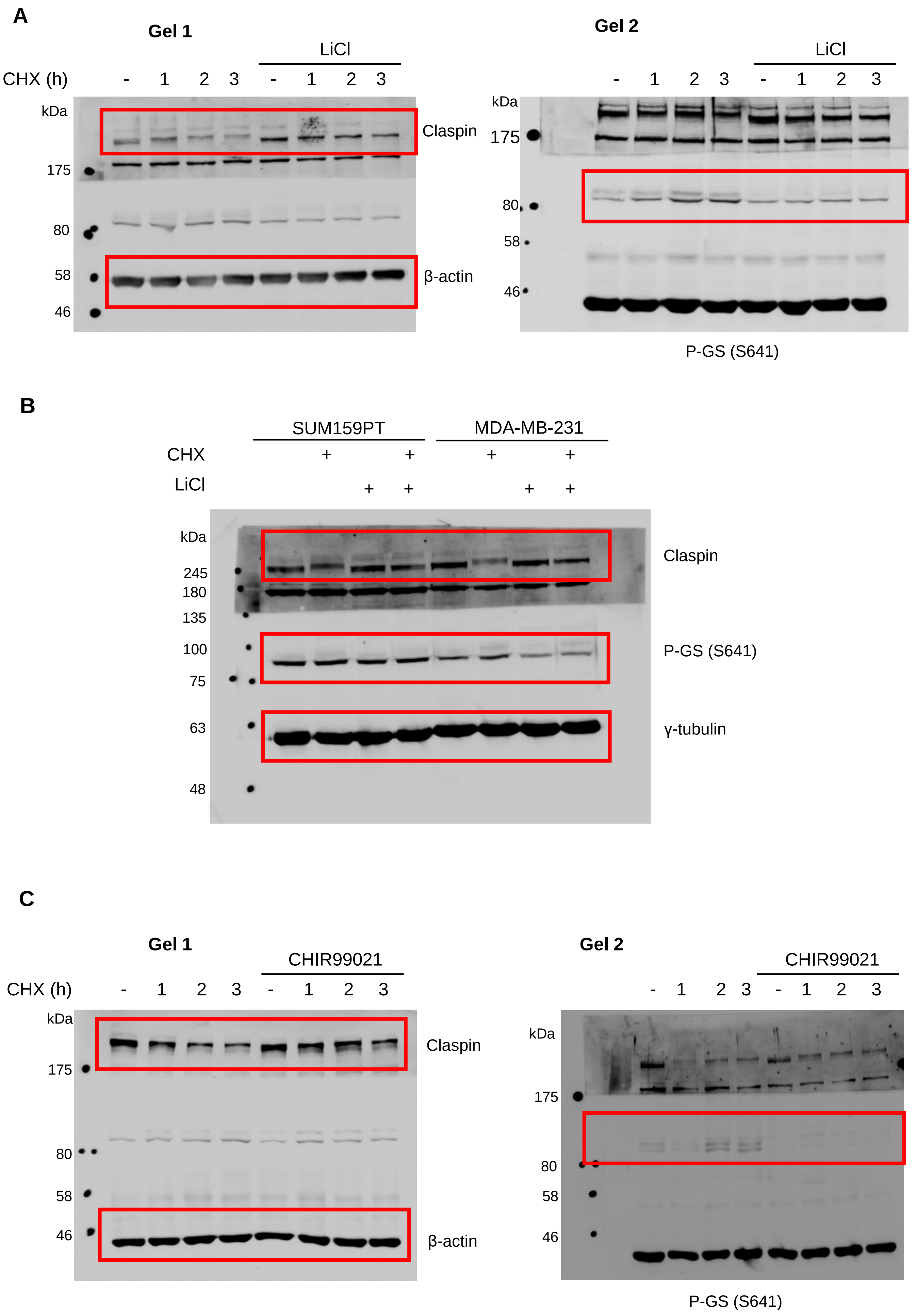
A
Gel 1
LiCl
CHX (h)
- 1 2 3 - 1 2 3
kDa
175
80
58
46
Claspin
β-actin
Gel 2
LiCl
- 1 2 3 - 1 2 3
kDa
175
80
58
46
P-GS (S641)
B
SUM159PT
MDA-MB-231
CHX
LiCl
+ + + +
+ + + +
kDa
245
180
135
100
75
63
48
Claspin
P-GS (S641)
γ-tubulin
C
Gel 1
CHIR99021
CHX (h)
- 1 2 3 - 1 2 3
kDa
175
80
58
46
Claspin
β-actin
Gel 2
CHIR99021
- 1 2 3 - 1 2 3
kDa
175
80
58
46
P-GS (S641)

Figure 1

D

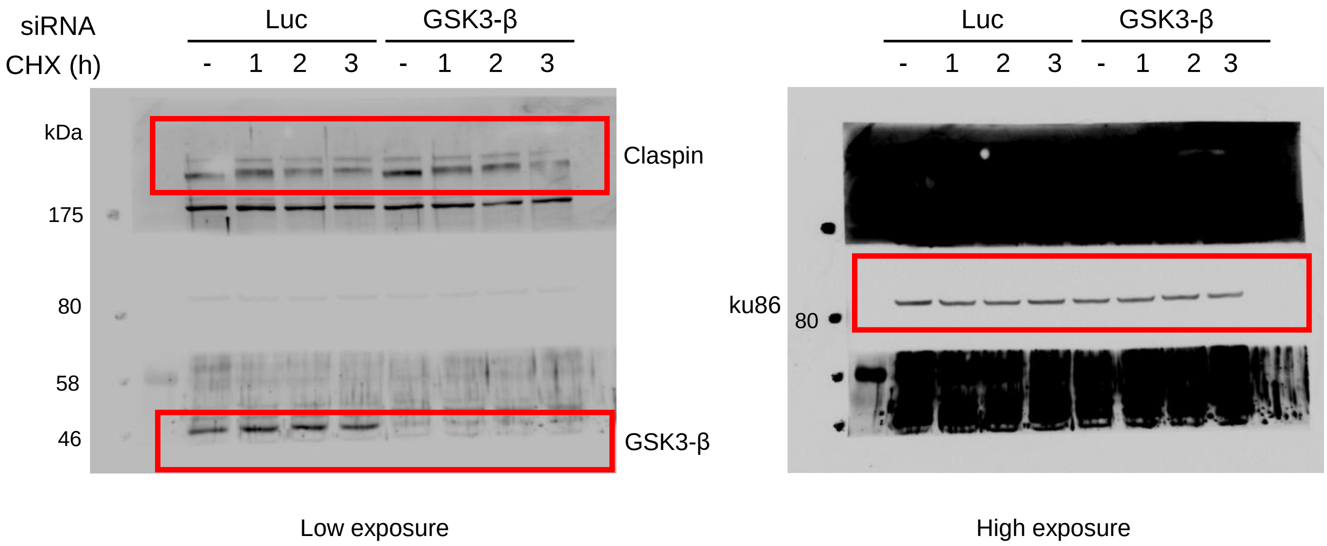

siRNA
CHX (h)
Luc
GSK3-β
- 1 2 3 - 1 2 3
kDa
175
80
58
46
Claspin
GSK3-β
Low exposure
Luc
GSK3-β
- 1 2 3 - 1 2 3
ku86
80
High exposure

E

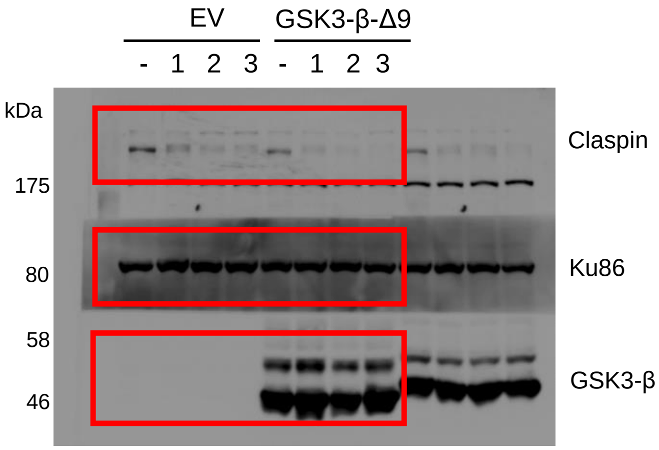

EV
GSK3-β-Δ9
- 1 2 3 - 1 2 3
kDa
175
80
58
46
Claspin
Ku86
GSK3-β

Figure 2

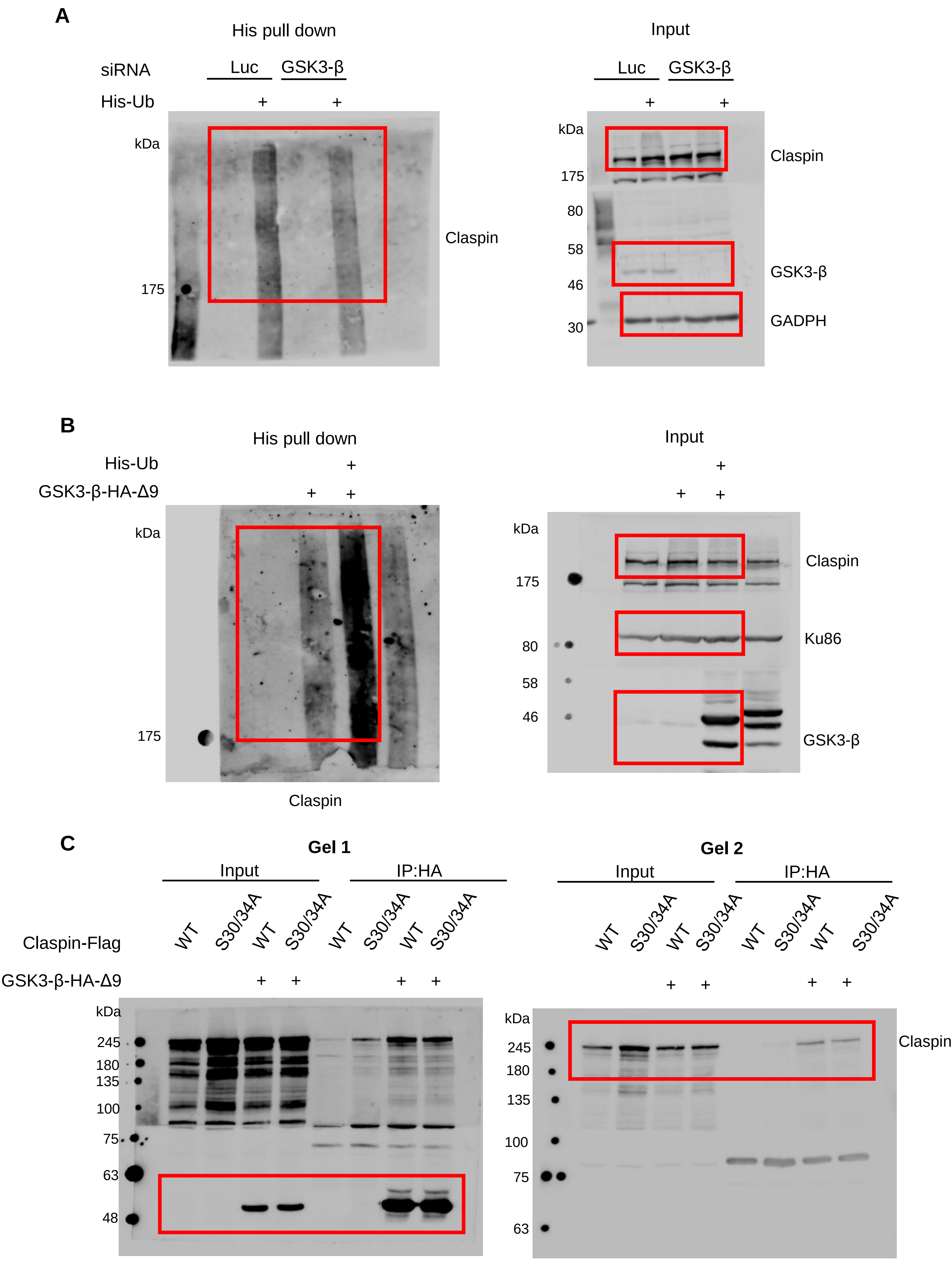
A
His pull down
siRNA
Luc
GSK3-β
His-Ub
+
+
kDa
175
Claspin
Input
Luc
GSK3-β
+
+
kDa
175
80
58
46
30
Claspin
GSK3-β
GADPH
B
His pull down
His-Ub
GSK3-β-HA-Δ9
kDa
175
Claspin
Input
kDa
175
80
58
46
Claspin
Ku86
GSK3-β
C
Gel 1
Input
IP:HA
Claspin-Flag
WT
S30/34A
GSK3-β-HA-Δ9
kDa
245
180
135
100
75
63
48
Gel 2
Input
IP:HA
kDa
245
180
135
100
75
63
Claspin

Figure 2

D

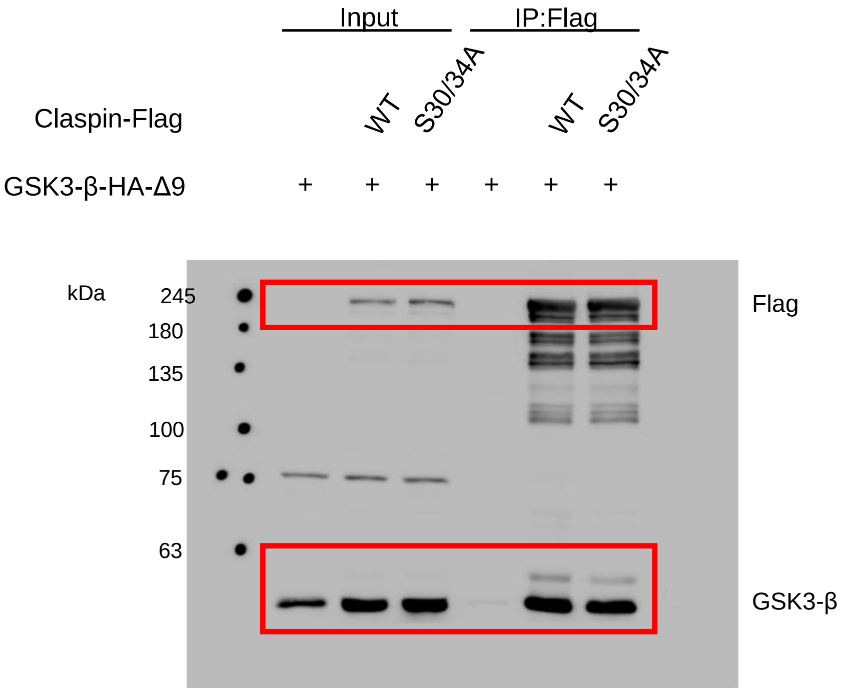

Input
IP:Flag
Claspin-Flag
WT
S30/34A
WT
S30/34A
GSK3-β-HA-Δ9
+ + + + + +
kDa
245
180
135
100
75
63
Flag
GSK3-β

E

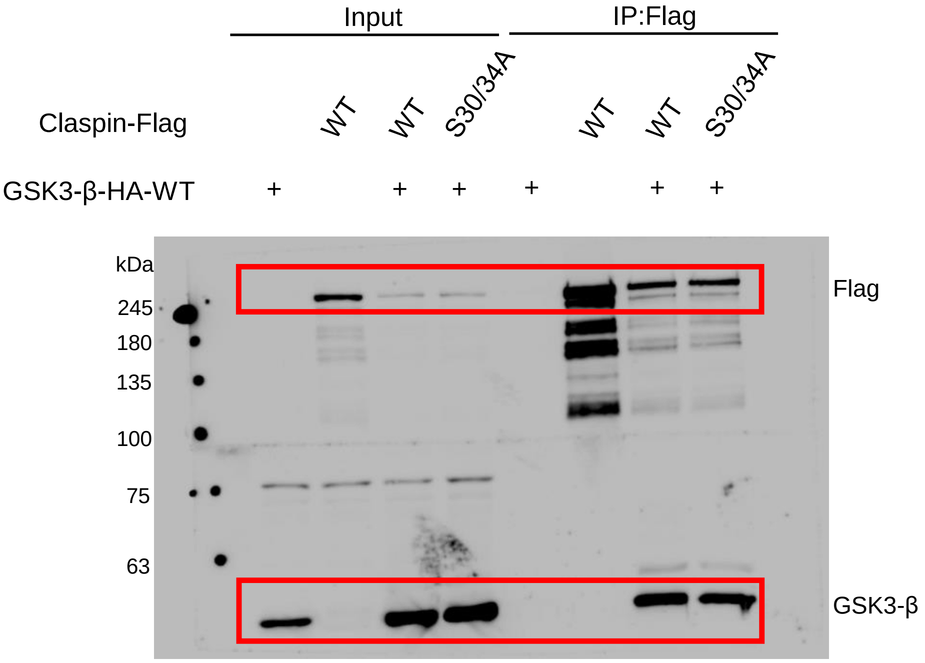

Input
IP:Flag
Claspin-Flag
WT
WT
S30/34A
WT
WT
S30/34A
GSK3-β-HA-WT
+ + + + + +
kDa
245
180
135
100
75
63
Flag
GSK3-β

Figure 3

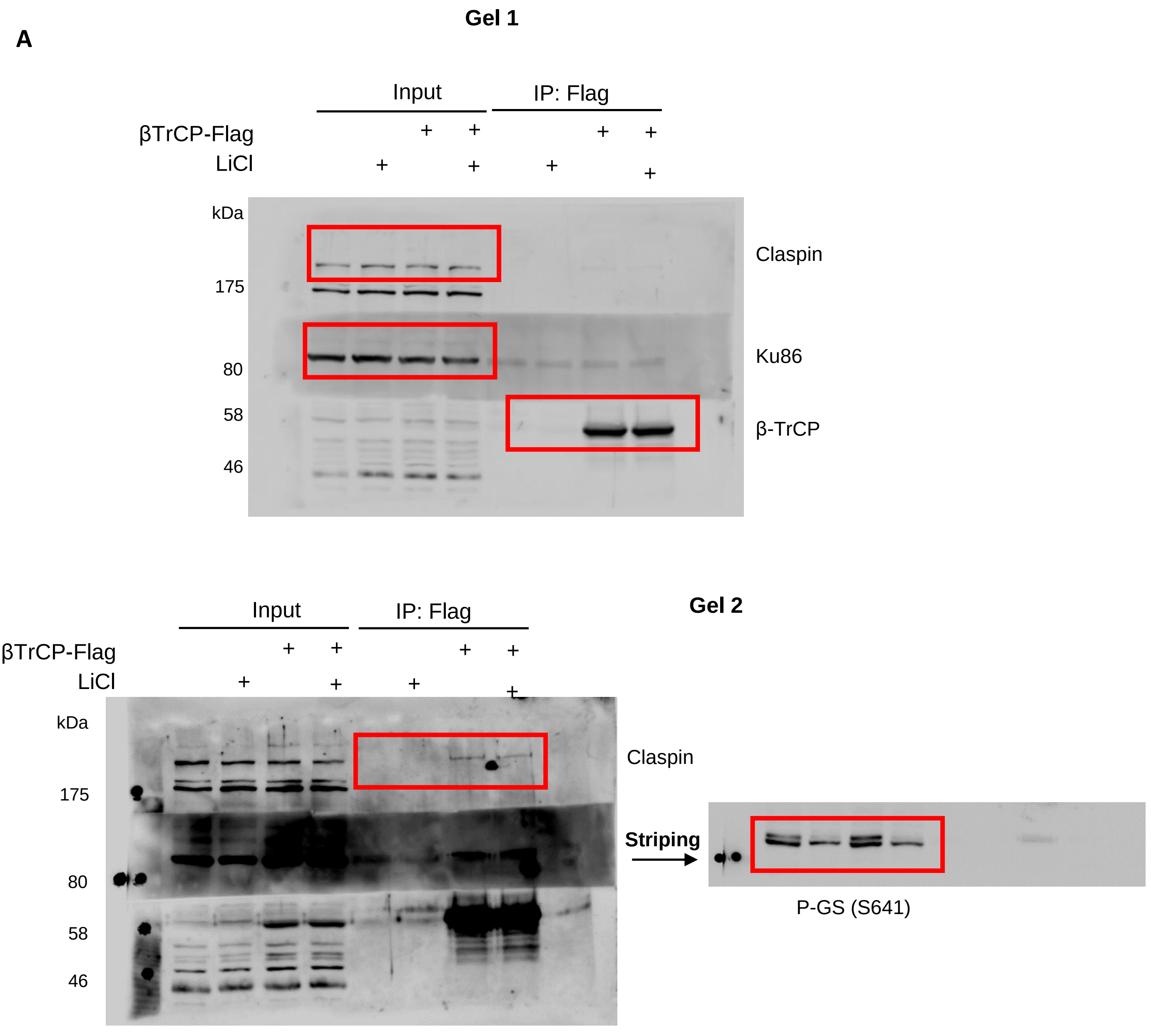

A
Gel 1
Input
IP: Flag
βTrCP-Flag
LiCl
kDa
175
80
58
46
Claspin
Ku86
β-TrCP
Gel 2
Input
IP: Flag
βTrCP-Flag
LiCl
kDa
175
80
58
46
Claspin
Striping
P-GS (S641)

Figure 3

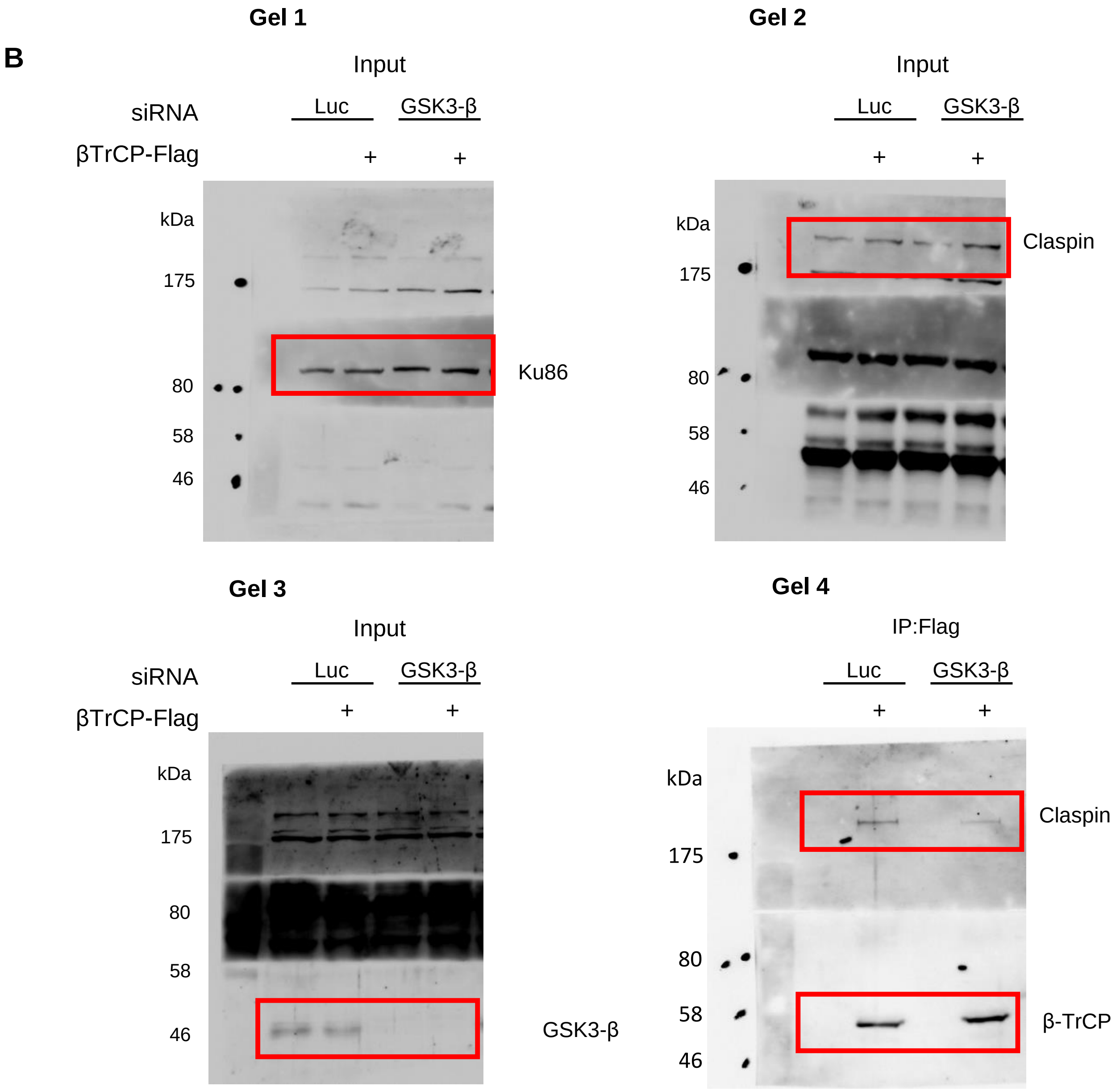
B
Gel 1
Input
siRNA
Luc
GSK3-β
βTrCP-Flag
+
+
kDa
175
80
58
46
Ku86
Gel 2
Input
Luc
GSK3-β
+
+
kDa
175
80
58
46
Claspin
Gel 3
Input
siRNA
Luc
GSK3-β
βTrCP-Flag
+
+
kDa
175
80
58
46
GSK3-β
Gel 4
IP:Flag
Luc
GSK3-β
+
+
kDa
175
80
58
46
Claspin
β-TrCP

Figure 3

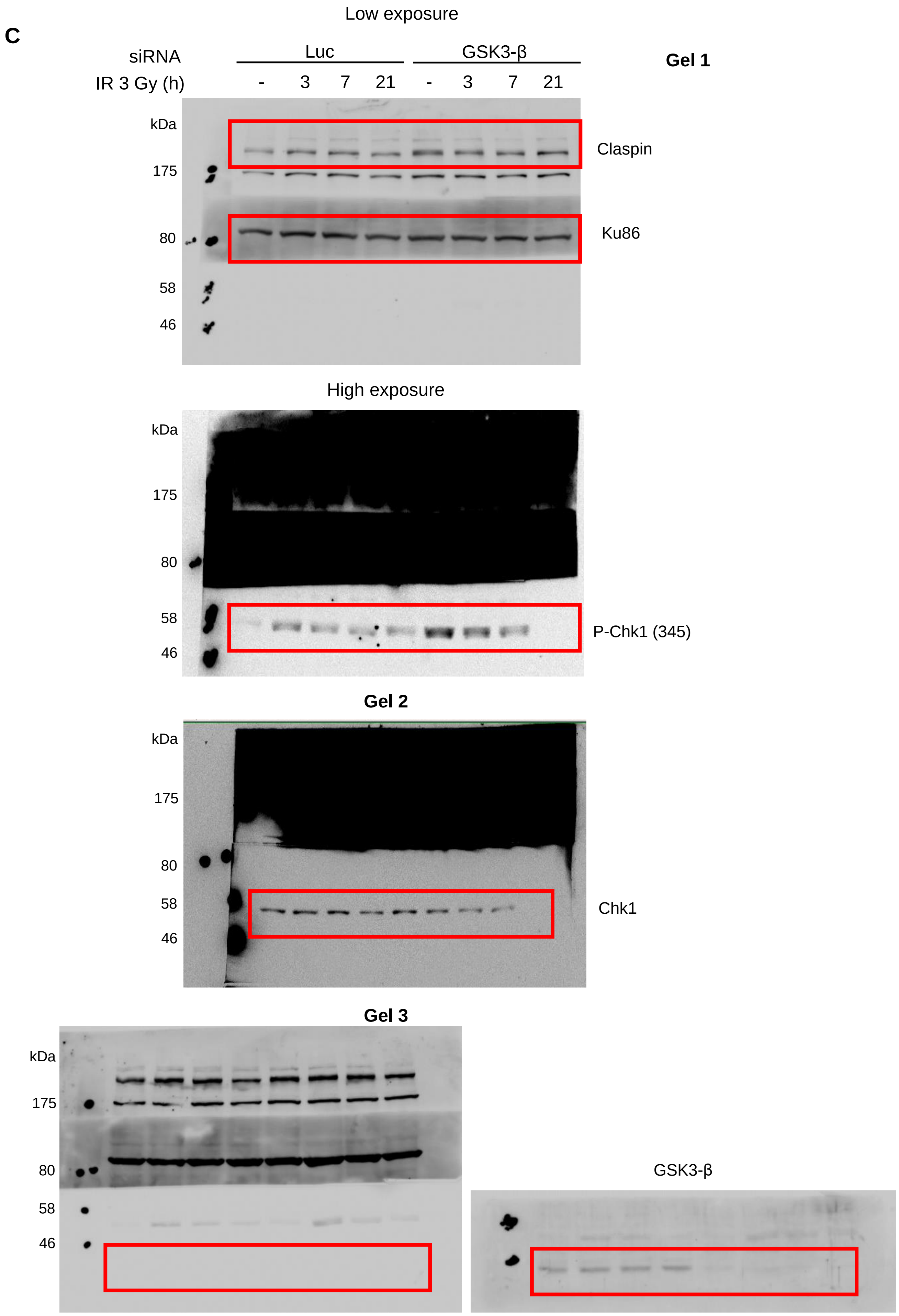
C
Low exposure
siRNA
Luc
GSK3-β
IR 3 Gy (h)
- 3 7 21 - 3 7 21
Gel 1
kDa
175
80
58
46
Claspin
Ku86
High exposure
kDa
175
80
58
46
P-Chk1 (345)
Gel 2
kDa
175
80
58
46
Chk1
Gel 3
kDa
175
80
58
46
GSK3-β

Figure 3

D

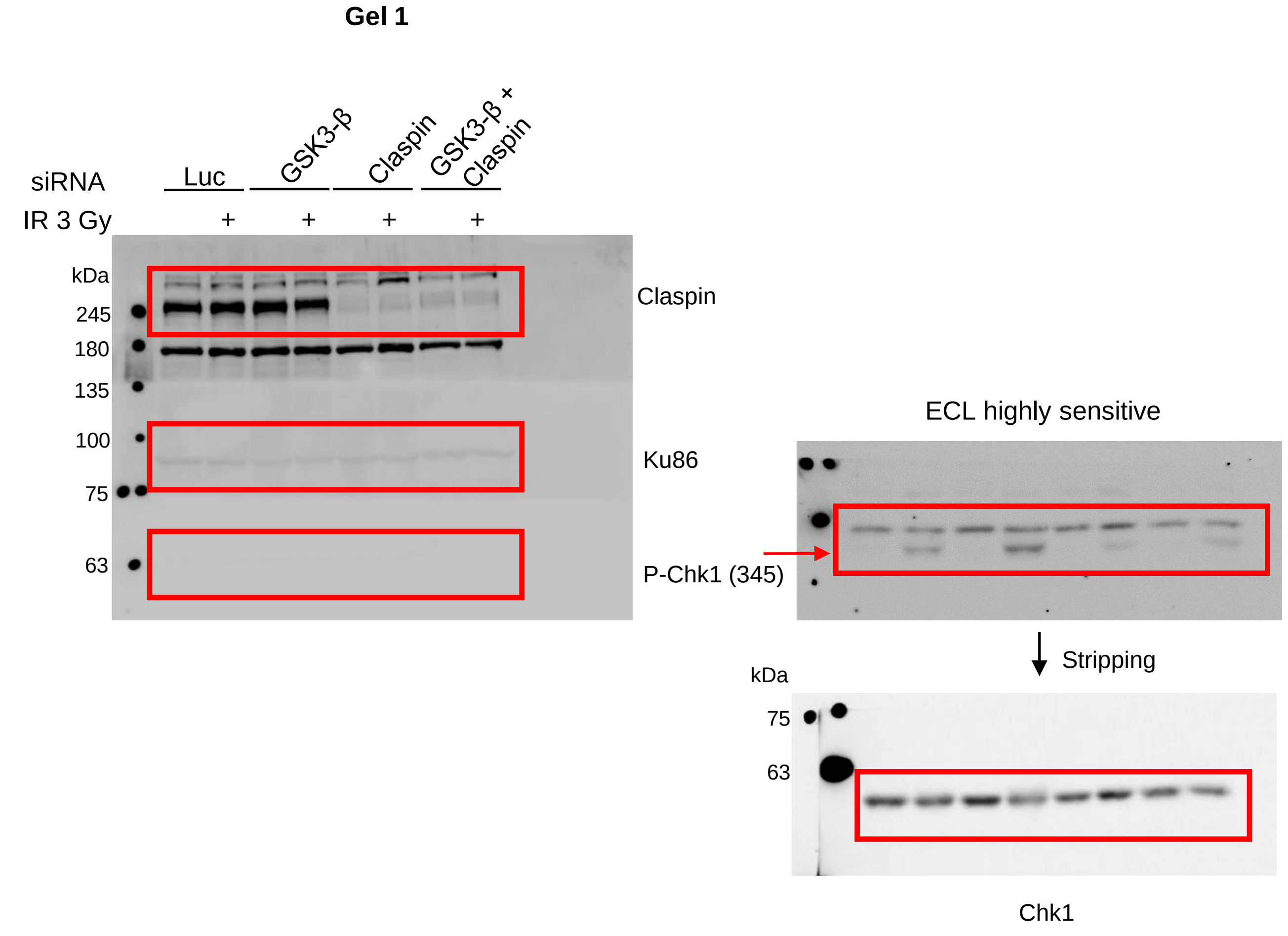
Gel 1
siRNA
Luc
GSK3-β
Claspin
GSK3-β + Claspin
IR 3 Gy
+
+
+
+
kDa
245
180
135
100
75
63
Claspin
Ku86
P-Chk1 (345)
ECL highly sensitive
Stripping
kDa
75
63
Chk1

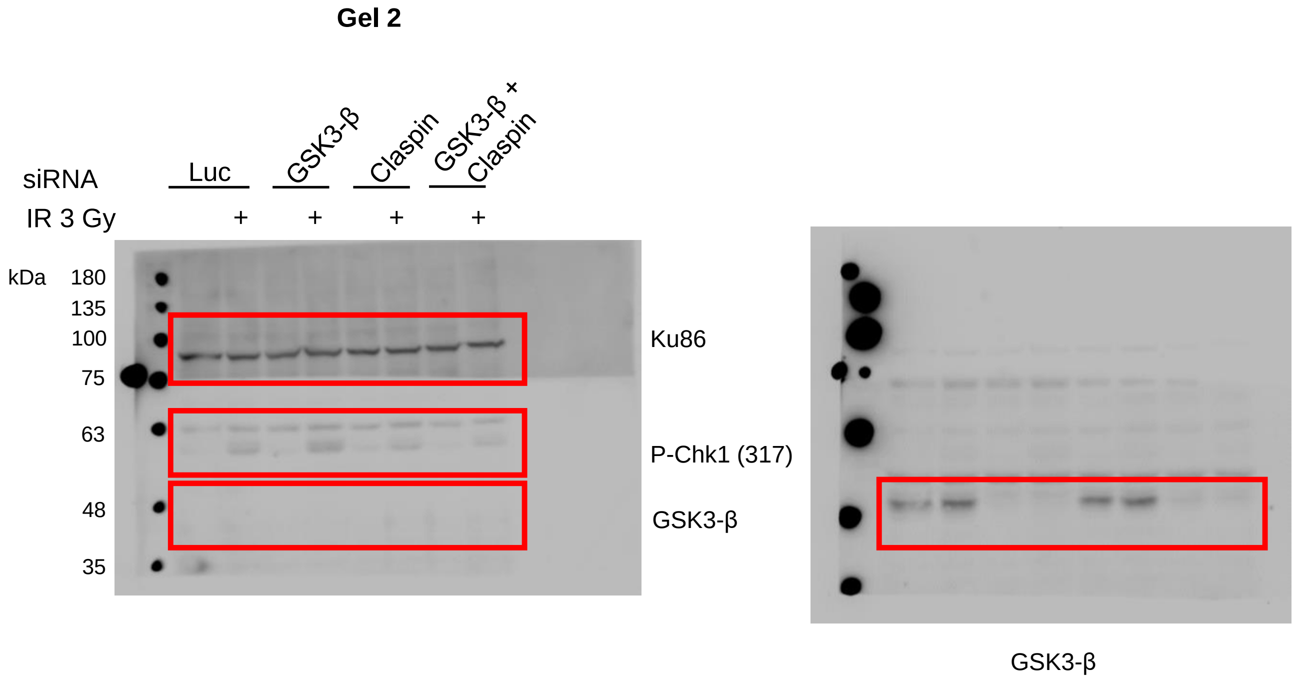
Gel 2
siRNA
Luc
GSK3-β
Claspin
GSK3-β + Claspin
IR 3 Gy
+
+
+
+
kDa
180
135
100
75
63
48
35
Ku86
P-Chk1 (317)
GSK3-β
GSK3-β

Figure 4

A

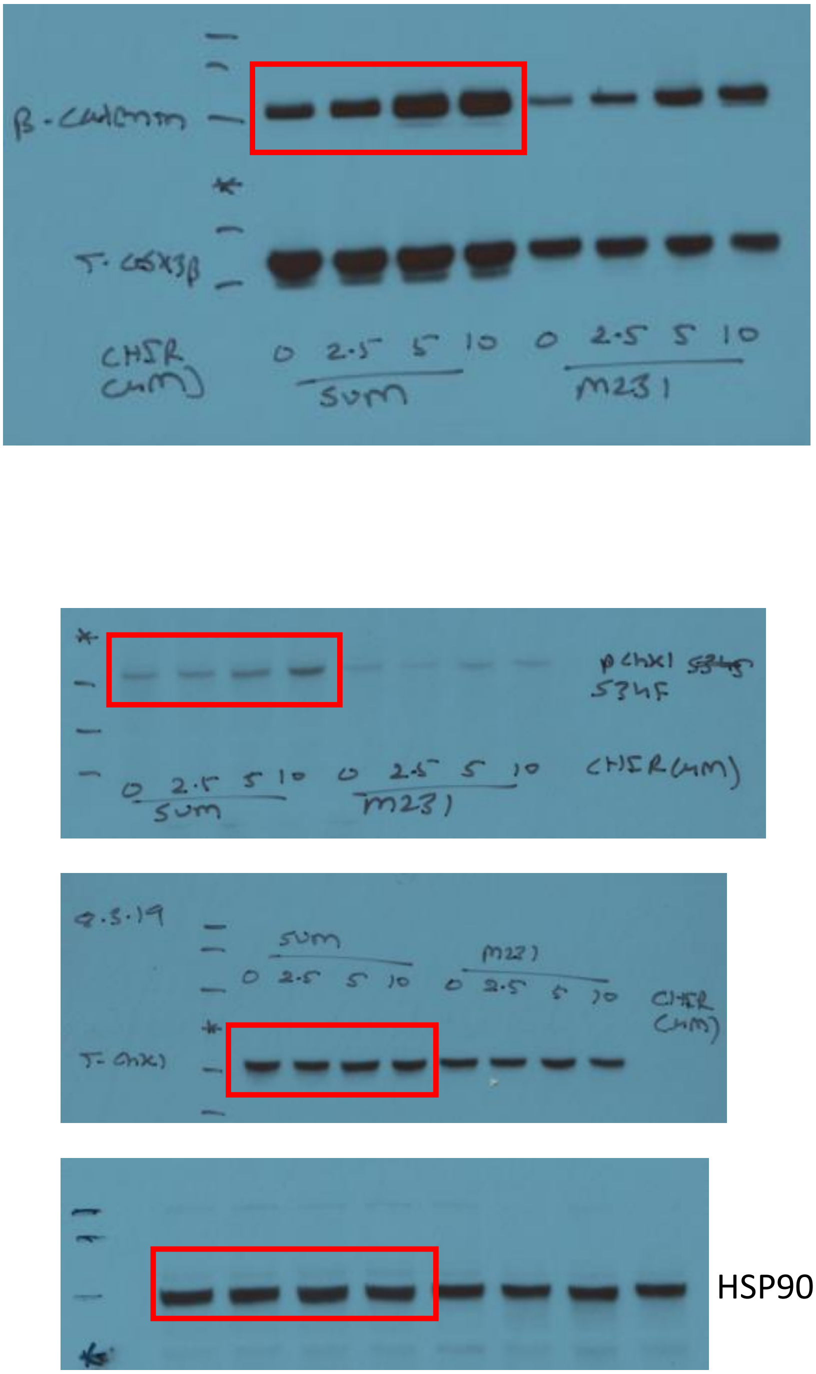

HSP90

CHIR (µM) 0 2.5 5 10

SUM159PT

Figure 4

A

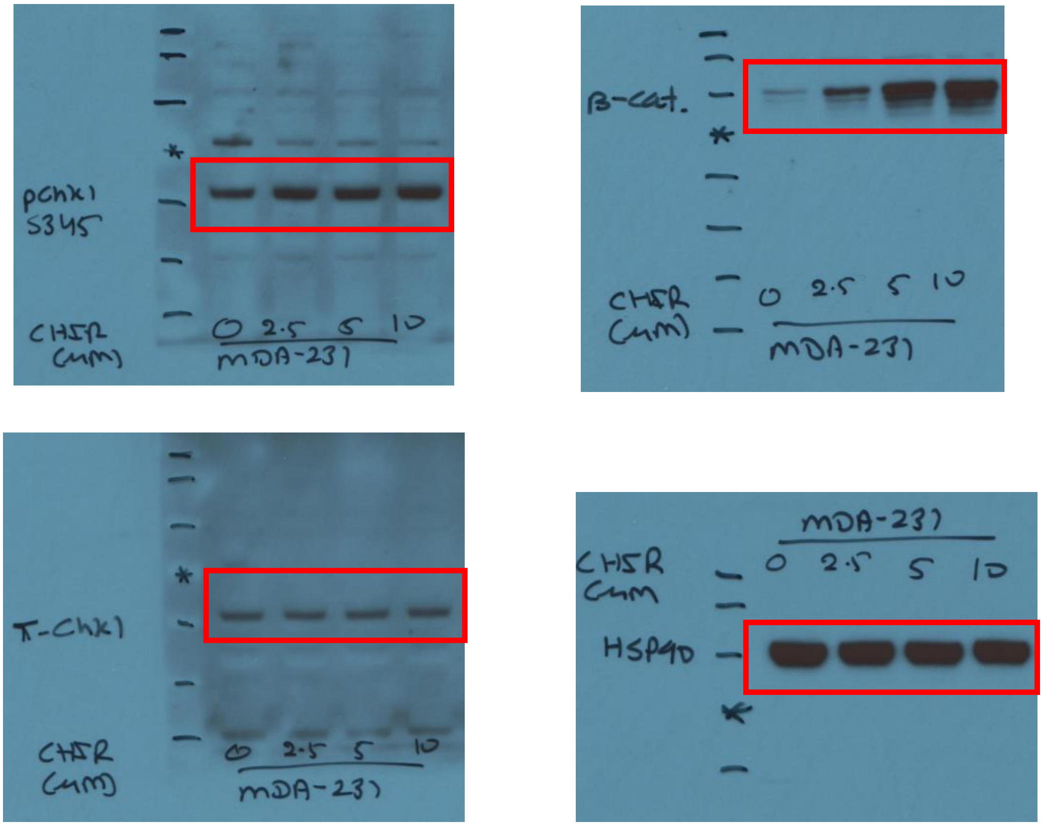

Figure 4

D

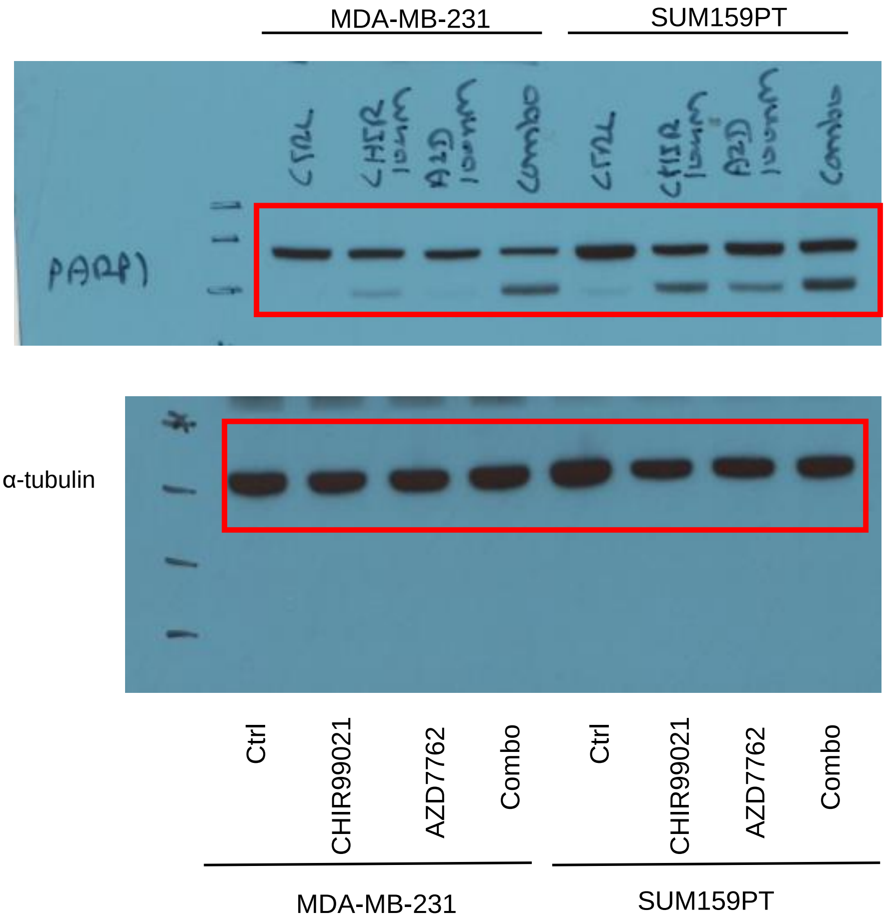
MDA-MB-231
SUM159PT
PARP1
α-tubulin
Ctrl
CHIR99021
AZD7762
Combo
Ctrl
CHIR99021
AZD7762
Combo
MDA-MB-231
SUM159PT

Supplemental data

Sup. Fig. 1

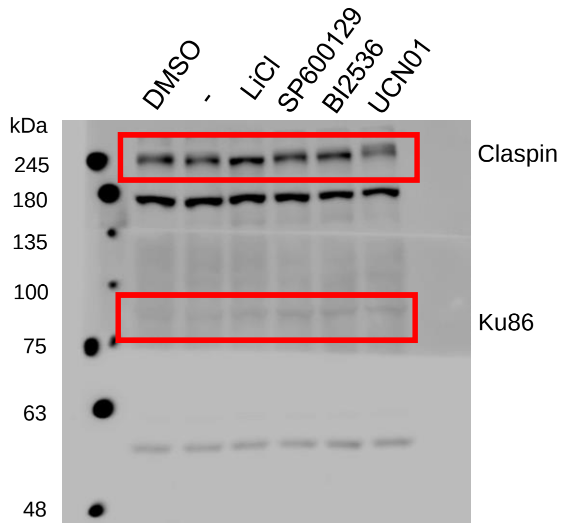

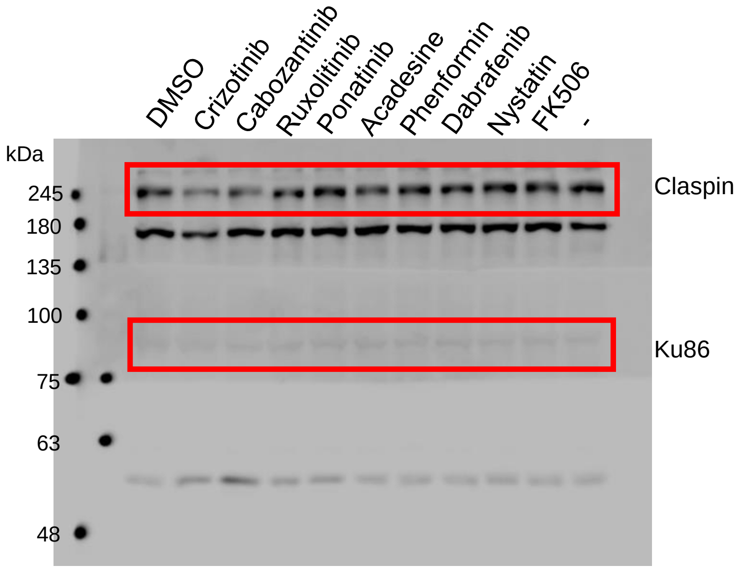

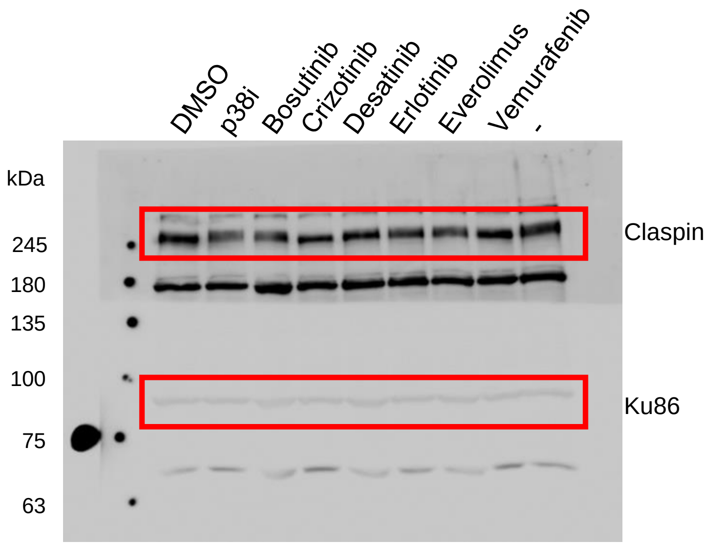

Supplemental data

Sup. Fig. 2A

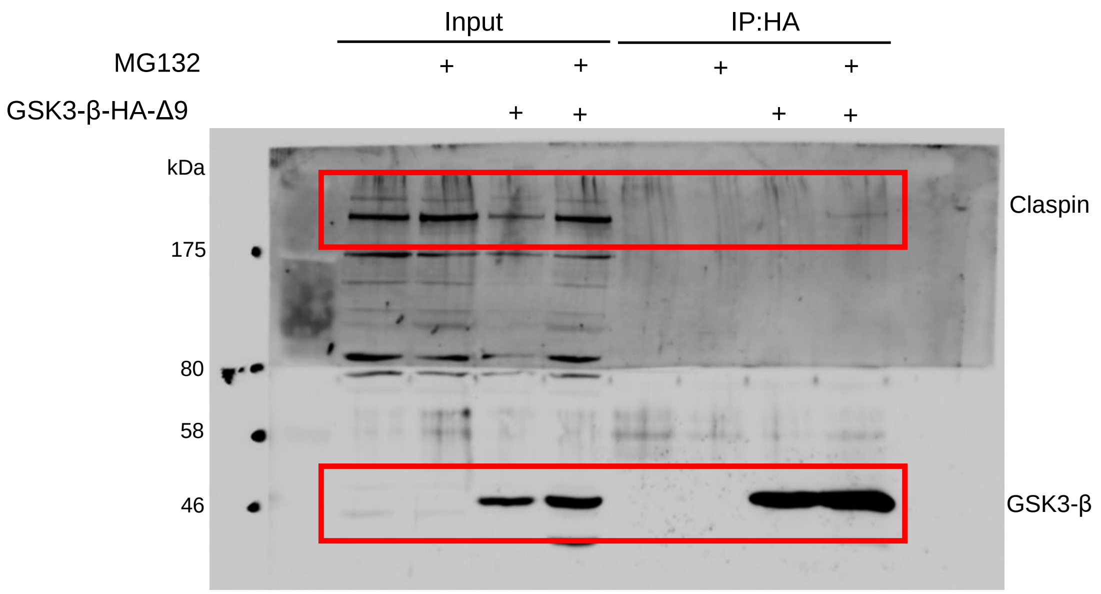

Sup. Fig. 2B

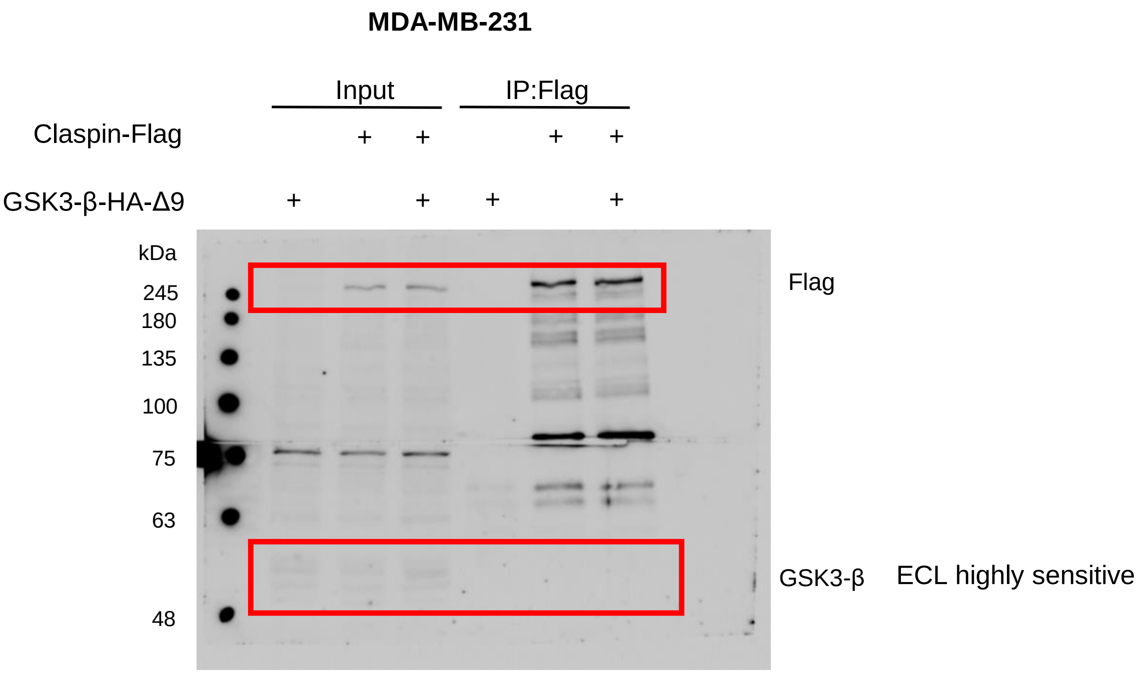

Supplemental data

Sup. Fig. 2C

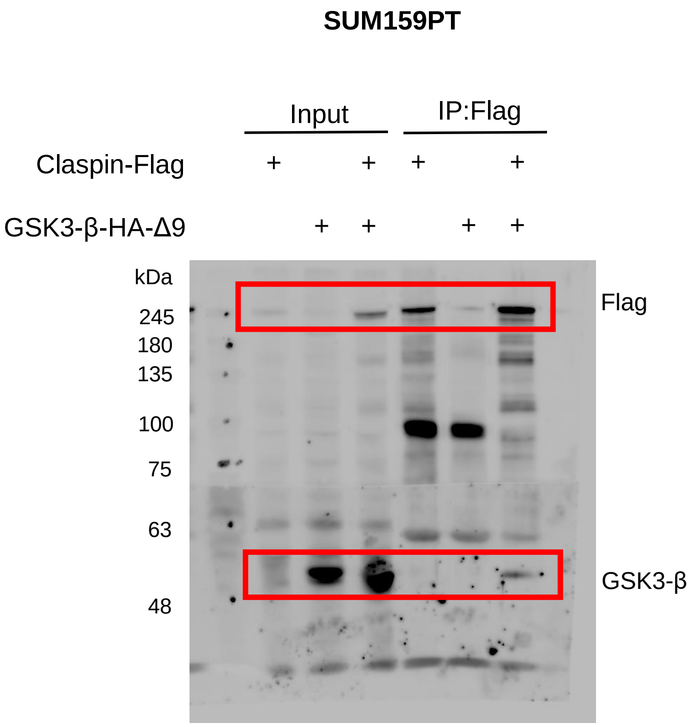

Supplement: Supplementary file 1 [file cancers-11-01073-s001.zip › Whole blots original pictures.pdf]
